# TDE-3: an improved prior for optical flow computation in spiking neural networks

**DOI:** 10.3389/fnins.2025.1667541

**Published:** 2025-11-03

**Authors:** Matthew Yedutenko, Federico Paredes-Vallés, Lyes Khacef, Guido de Croon

**Affiliations:** ^1^Micro Air Vehicle Laboratory, Delft University of Technology, Delft, Netherlands; ^2^Sony Semiconductor Solutions Europe, Sony Europe B.V., Zurich, Switzerland

**Keywords:** edge processing, brain-inspired computing, spiking neural networks, time difference encoders, motion detection, optical flow

## Abstract

Motion detection is a primary task required for robotic systems to perceive and navigate in their environment. Proposed in the literature bioinspired neuromorphic Time-Difference Encoder (TDE-2) combines event-based sensors and processors with spiking neural networks to provide real-time and energy-efficient motion detection through extracting temporal correlations between two points in space. However, on the algorithmic level, this design leads to a loss of direction-selectivity of individual TDEs in textured environments. In the present work, we propose an augmented 3-point TDE (TDE-3) with additional inhibitory input that makes TDE-3 direction-selectivity robust in textured environments. We developed a procedure to train the new TDE-3 using backpropagation through time and surrogate gradients to linearly map input velocities into an output spike count or an Inter-Spike Interval (ISI). Using synthetic data, we compared training and inference with spike count and ISI with respect to changes in stimuli dynamic range, spatial frequency, and level of noise. ISI turns out to be more robust toward variation in spatial frequency, whereas the spike count is a more reliable training signal in the presence of noise. We conducted an in-depth quantitative investigation of optical flow coding with TDE and compared TDE-2 vs. TDE-3 in terms of energy efficiency and coding precision. The results show that at the network level, both detectors show similar precision (20° angular error, 88% correlation with the truth of the ground). However, due to the more robust direction selectivity of individual TDEs, the TDE-3 based network spikes less and is hence more energy efficient. Reported precision is on par with model-based methods but the spike-based processing of the TDEs provides allows more energy-efficient inference with neuromorphic hardware. Additionally, we also employed TDE-2 and TDE-3 to estimate ego-motion and showed results competitive with those achieved by neural networks with 1.5 × 10^5^ parameters.

## 1 Introduction

Event-based cameras are the dawn of the brave new world of ultra-efficient neuromorphic hardware. Taking inspiration from biological retinas, these cameras transmit only temporal changes in brightness using asynchronous events (Lichtsteiner et al., 2008). As a result, they require >10^2^× less energy and memory than traditional frame-based cameras, while having 10^4^× higher dynamic range and up to 10^4^× shorter latency (Gallego et al., 2022), which is particularly handy in robotics applications. Under constant illumination, temporal changes in brightness are generated by motion. Therefore, optical flow estimation is one of, if not the main task in event-based vision.

**Figure 1:**
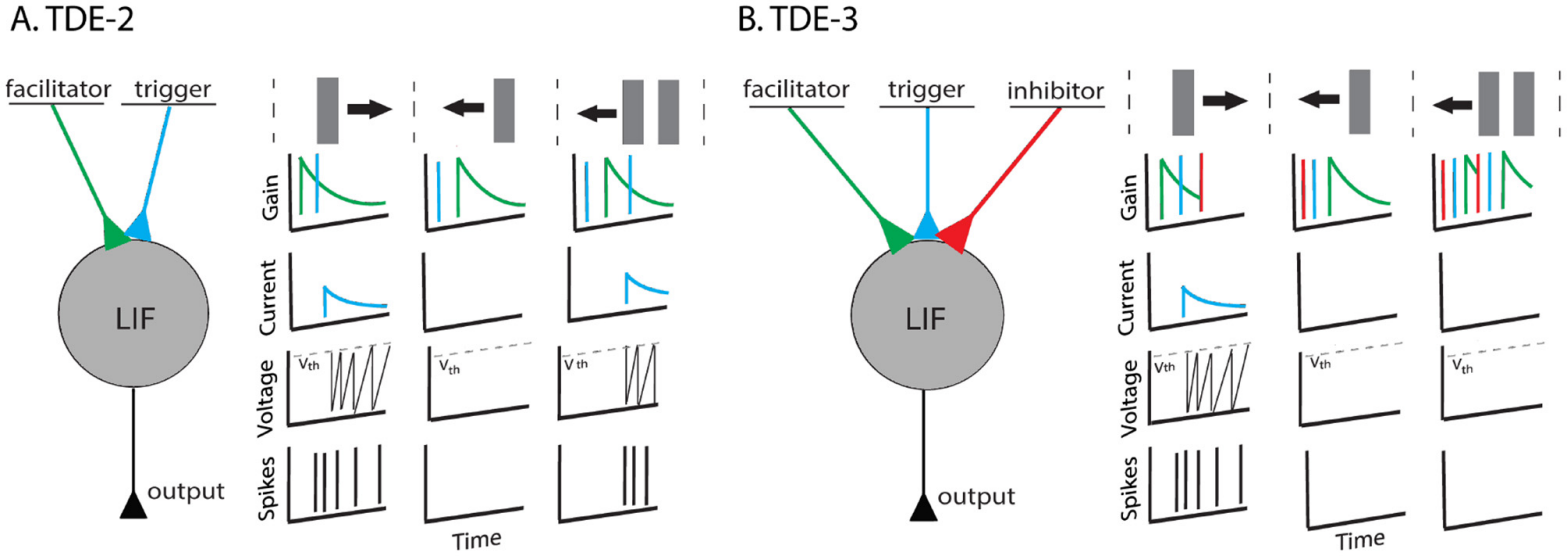
TDE-2 and TDE-3. **(A)** Left-to-right tuned 2-point TDE. It has two compartments: the facilitator and the trigger. When a stimulus moves to the right the trigger is activated after the facilitator and the neuron fires. However, in the gain, there is residual activity. Thus, when multiple textures move left (or orthogonally) the detector loses direction-selectivity **(B)**. Left-to-right tuned 3-point TDE. It has three compartments. Input to the inhibitor resets the gain to zero and removes residual activity. Therefore, direction-selectivity is retained.

The efficiency of event-based processing in the estimation of optical flow is especially well utilized by spiking neural networks (SNNs)—computational abstractions of biological neurons that transmit signals in an all-or-none spiking fashion only when the internal neuronal activation reaches a certain threshold (Maass, 1997b) (Figure 1). Indeed, asynchronous all-or-none spikes seem like a perfect match to asynchronous all-or-none events due to the similar nature of these signals. Studies showed that when it comes to optical flow, SNNs can perform on par (Hagenaars et al., 2021) or even better (Kosta and Roy, 2023; Cuadrado et al., 2023) than state-of-the-art traditional neural networks, while requiring up to 50-times fewer parameters (Kosta and Roy, 2023) and promising up to 10^3^ decrease in compute energy (Lee et al., 2020; Zhang et al., 2023). With the development of specially designated to processing SNNs neuromorphic processors (Davies et al., 2018), event-based optical flow estimation with SNNs reached a significant milestone of technological maturity—implementation for vision-based navigation onboard a drone in a fully neuromorphic pipeline (Paredes-Vallés et al., 2023).

Although theoretically SNNs are capable of performing a wide variety of tasks (McCulloch and Pitts, 1943; Hornik et al., 1989; Maass, 1997a,b), their actual performance strongly depends on the choice of proper priors such as neural network architecture and connectivity on the hardware and algorithmic levels. For example, in image classification tasks, the introduction of randomly initialized and non-trainable convolutional kernels improves the classification accuracy compared to fully connected networks (Frenkel et al., 2021) simply because convolutional kernels extract a local spatial pattern. Similarly, proper priors to extract spatio-temporal patterns could enhance algorithms of the optical flow estimation from event-based data.

A good computational prior for optical flow estimation from event-based data could be the correlation-based motion detector, which is ubiquitously employed in the animal kingdom (Clark and Demb, 2016). As its name suggests, this detector extracts local motion direction and velocity (i.e. optical flow) based on the temporal correlations between different points in space (Clark and Demb, 2016). Such correlation-based estimates were shown to closely approach the optimal way to extract motion signals in conditions with low Signal-to-Noise Ratio (SNR) (Sinha et al., 2021; Potters and Bialek, 1994). Event-based cameras, meanwhile, are prone to noisy outputs (Guo and Delbruck, 2023) due to the environment (low contrast and light intensity Brosch et al., 2015), the temperature Czech and Orchard (2016); Nozaki and Delbruck (2017), the junction leakage (Nozaki and Delbruck, 2017; Ding et al., 2023), the hardware limitations (manufacturing mismatch Lichtsteiner et al., 2008), and the very differential nature of processing which amplifies high-frequency noise (Ding et al., 2023; Brosch et al., 2015; Simoncelli, 1993). Given such low SNR output, a correlation-based motion detector is well-suited to process event-based data.

Bio-inspired correlation-based methods for optical flow estimation with SNNs generated long-lasting interest in event-based vision and neuromorphic computing communities with over a decade of relevant research on both software (Paredes-Valles et al., 2020; Barranco et al., 2015; Brosch et al., 2015; Zheng et al., 2023; Orchard et al., 2013) and hardware levels (Brosch and Neumann, 2016; Milde et al., 2018; Schoepe et al., 2024; Giulioni et al., 2016; D'Angelo et al., 2020; Gutierrez-Galan et al., 2022; Haessig et al., 2018; Chiavazza et al., 2023; Sarpeshkar et al., 1996; Kramer, 1996; Etienne-Cummings et al., 1993). In particular, for our study, we are interested in the inspired by the classical model of the insect motion detector (Reichardt, 1961) Time-Difference Encoder (TDE) (Milde et al., 2018) due to its circuit and algorithmic simplicity (Milde et al., 2018). The TDE detects motion by comparing signals at two points in space: input to the facilitator provides a time-decaying gain, whereas input to the trigger converts gain to an input current impulse to a Current-Based Leaky Integrate-and-Fire (CuBa LIF) neuron, which integrates it in its membrane potential and converts it into a firing activity as shown in Figure 1. The TDE tuning to the preferred direction of motion (PD) is achieved since it produces output spikes only when the trigger event arrives after the facilitator one, as otherwise there is no gain to be converted to firing activity. The TDE was successfully implemented on FPGA Gutierrez-Galan et al. (2022), digital neuromorphic hardware such as SpiNNaker (D'Angelo et al., 2020), Loihi^1^ and Loihi 2 (Chiavazza et al., 2023), and custom mixed-signal neuromorphic hardware (Milde et al., 2018). Its potential was demonstrated in robotics applications for collision avoidance Milde et al. (2018); Schoepe et al. (2024) and estimation of ego-motion (Greatorex et al., 2025). The strength of the underlying computational primitive was also explored in tasks that are not related to vision, such as spotting keywords (Nilsson et al., 2023).

Despite its appeal, the classical two-point TDE (TDE-2) has a notable deficiency: in highly textured environments, it loses direction selectivity because motion in a non-preferred direction provides the TDE with residual activity in the gain compartment, which can be subsequently triggered by another edge moving in the non-preferred direction as shown in Figure 1. Given that successful event-based navigation requires events that are produced by the apparent motion of textures (Paredes-Vallés et al., 2023), loss of direction-selectivity makes classical TDE-2 unsuitable for vision-based navigation tasks that require precision in optical flow estimation. Of course, this loss in direction-selectivity can be partially compensated by either max pooling (Chiavazza et al., 2023), Winner-Take-All mechanism (Milde et al., 2018; Schoepe et al., 2024) or subtraction of the velocities estimated by opposingly tuned detectors. Yet in that case, the energy-efficiency of the circuitry would still be obstructed as individual TDEs would still respond to the non-preferred motion.

To solve this problem, we propose to follow recent findings in neuroscience (Haag et al., 2016, 2017) and to augment the TDE with a third, inhibitory input. While the classical model of elementary motion sensitivity in insects (Reichardt, 1961; Clark and Demb, 2016) estimated temporal correlations from two spatial locations, (Haag et al., 2016, 2017) showed that insect elementary motion detectors (so-called T4 and T5 cells) pools signals from three spatial locations with one of them being inhibitory and activated by the motion in ND. In our implementation, activation of this inhibitory input resets the gain to zero and eliminates the residual activity to retain the TDE direction-selectivity (Figure 1). Consequently, even in highly textured environments, individual TDEs retain direction selectivity, and the entire circuit spikes much less, becoming more energy-efficient. We propose to call this detector TDE-3.

**Figure 2:**
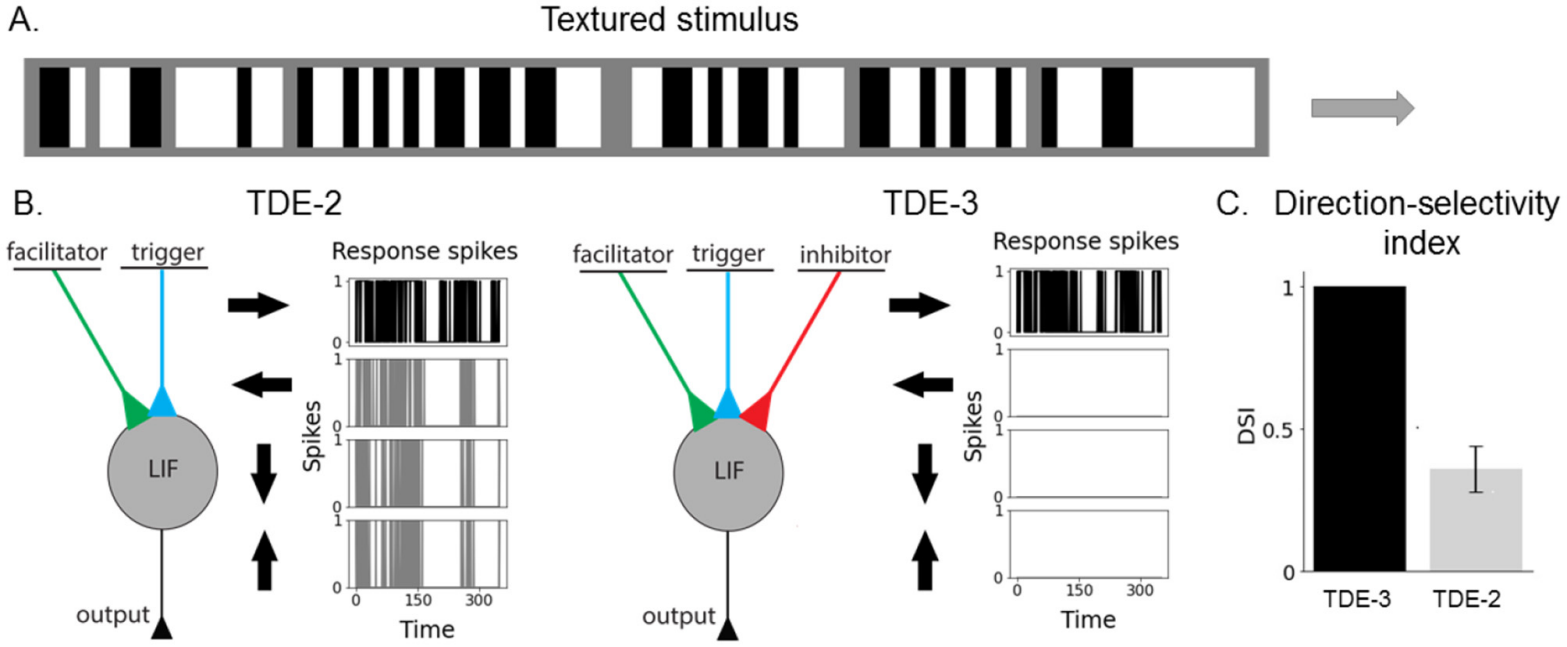
Augmented 3-point TDE retains direction-selectivity in a textured environment. **(A)** Visual stimulus composed of vertical bars. The bars had three light intensity levels: white, gray, and black. The size of the texture along the motion axis was 80 pixels and 3 pixels along the orthogonal direction. We employed 5 velocities: 0.1 px/timestep, 0.2 px/timestep, 0.33 px/timestep, 0.5 px/timestep and 1 px/timestep. The motion direction (left-right, right-left, top-bottom, bottom-top) and velocity were randomly chosen for each stimulus example (2,000 examples per testing round, 400 testing rounds). To vary the “amount” of texture in stimuli we randomly varied the fraction of the gray bars from 0% to 80%. **(B)** TDE-2 and TDE-3 and their responses to stimuli moving in 4 cardinal directions. **(C)** Direction-selectivity index (fraction of spikes fired upon stimulus motion in PD).

**Figure 3:**
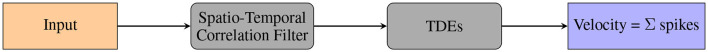
Pipeline for supervisory training of TDE with BPTT to linearly map velocities with spike-count based inference.

**Figure 4:**
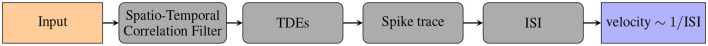
Pipeline for supervisory training of TDE with BPTT to linearly map velocities with ISI-based inference.

**Figure 5:**
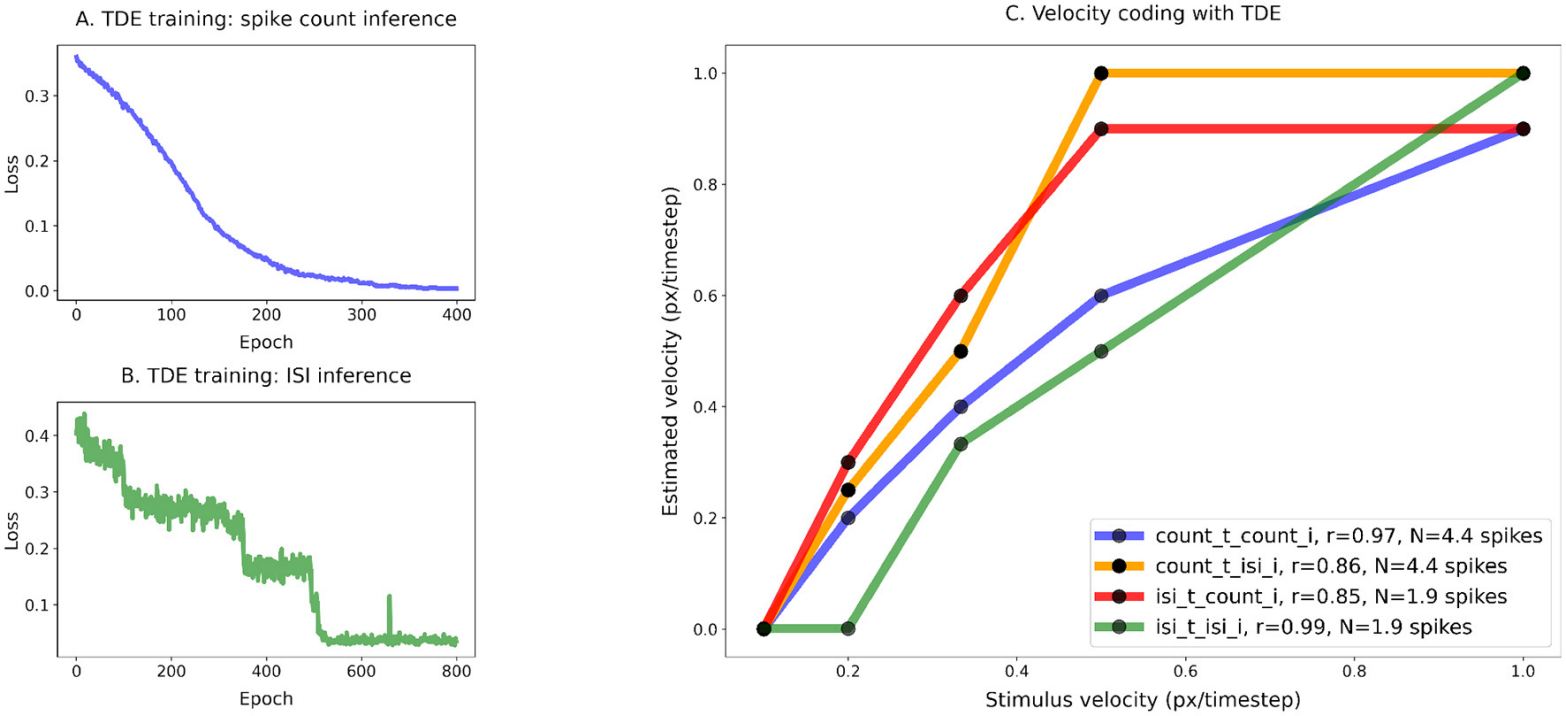
Training of the TDE-3: wide dynamic range, low resolution, one moving edge **(A)** Loss function for spike count-based inference, **(B)** loss function for ISI-based inference, **(C)** comparison of the velocity tuning curves. Blue—training with spike count, inference with spike count during the test; Orange—training with spike count, inference with ISI during the test; Green—training with ISI, inference with ISI during the test; Red—training with ISI, inference with spike count during the test.

**Figure 6:**
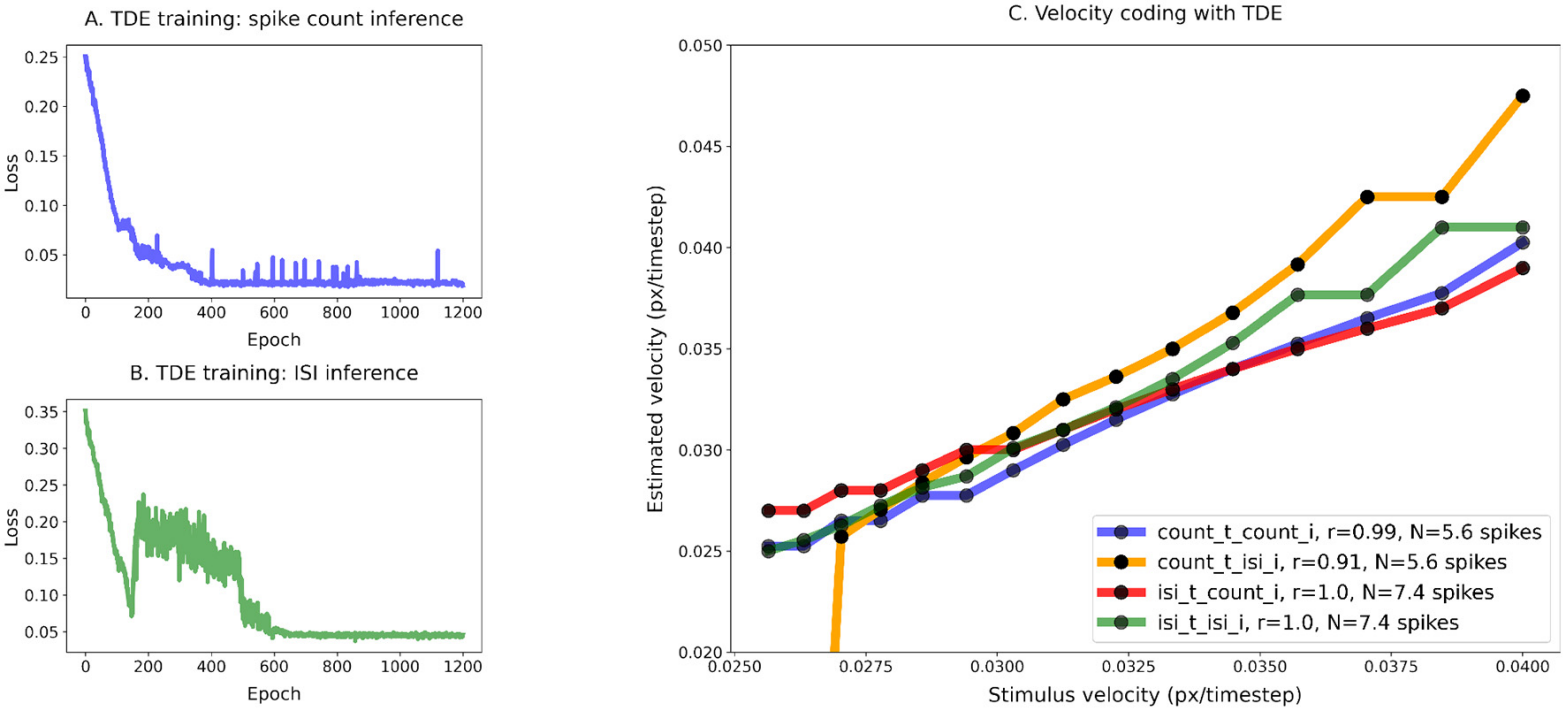
Training of the TDE-3: narrow dynamic range, high resolution, one moving edge. Training of the TDE-3: wide dynamic range, low resolution. **(A)** Loss function for spike count-based inference, **(B)** loss function for ISI-based inference, and **(C)** comparison of the velocity tuning curves. Blue—training with spike count, inference with spike count during the test; Orange—training with spike count, inference with ISI during the test; Green—training with ISI, inference with ISI during the test; Red—training with ISI, inference with spike count during the test.

**Figure 7:**
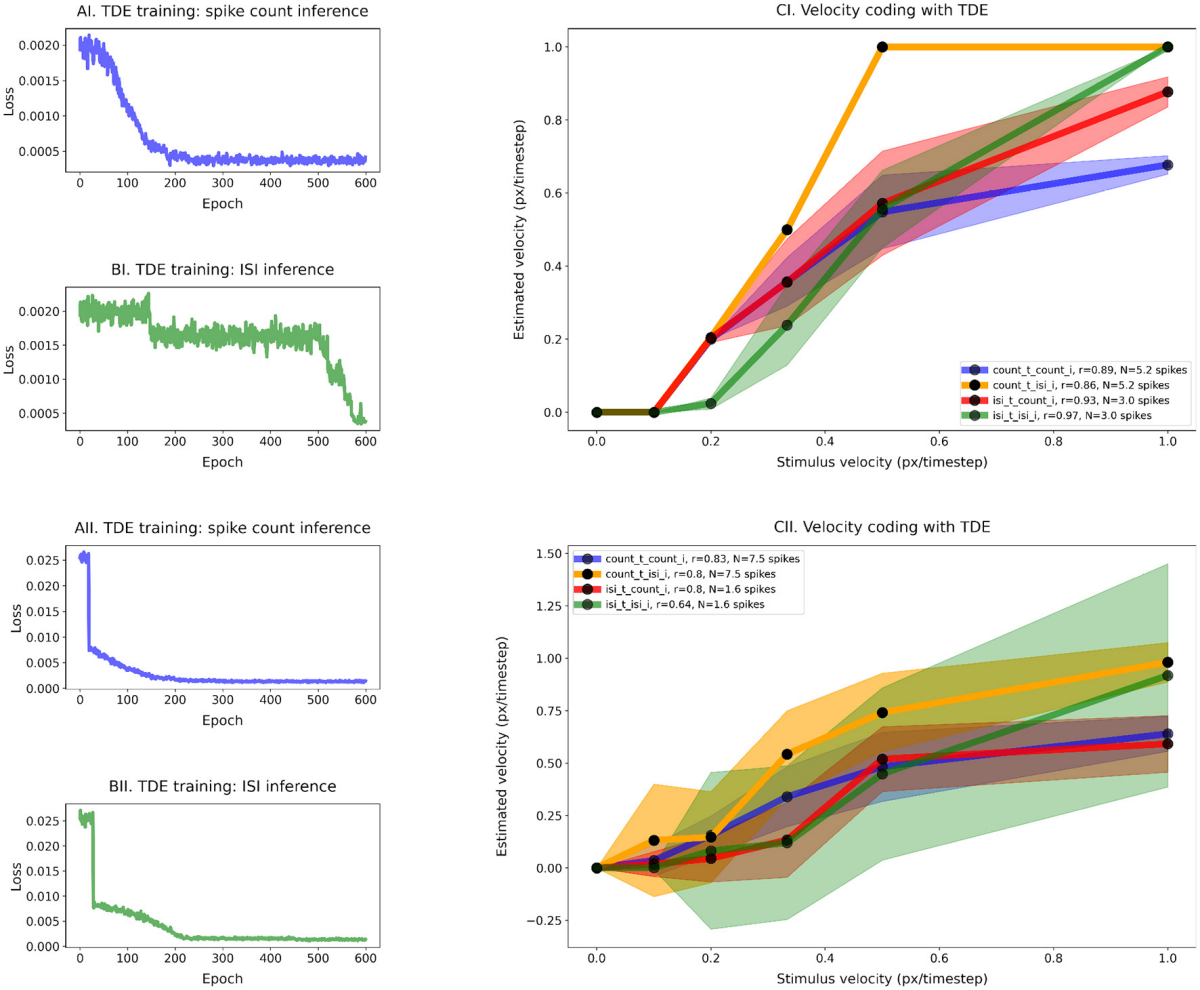
Training TDE: performance in the presence of noise. Top row—low noise, Bottom row—high noise. **(A)** Loss function for spike count-based inference, **(B)** loss function for ISI-based inference, and **(C)** comparison of the velocity tuning curves. Blue - training with spike count, inference with spike count during the test; Orange—training with spike count, inference with ISI during the test; Green—training with ISI, inference with ISI during the test; Red—training with ISI, inference with spike count during the test.

**Figure 8:**
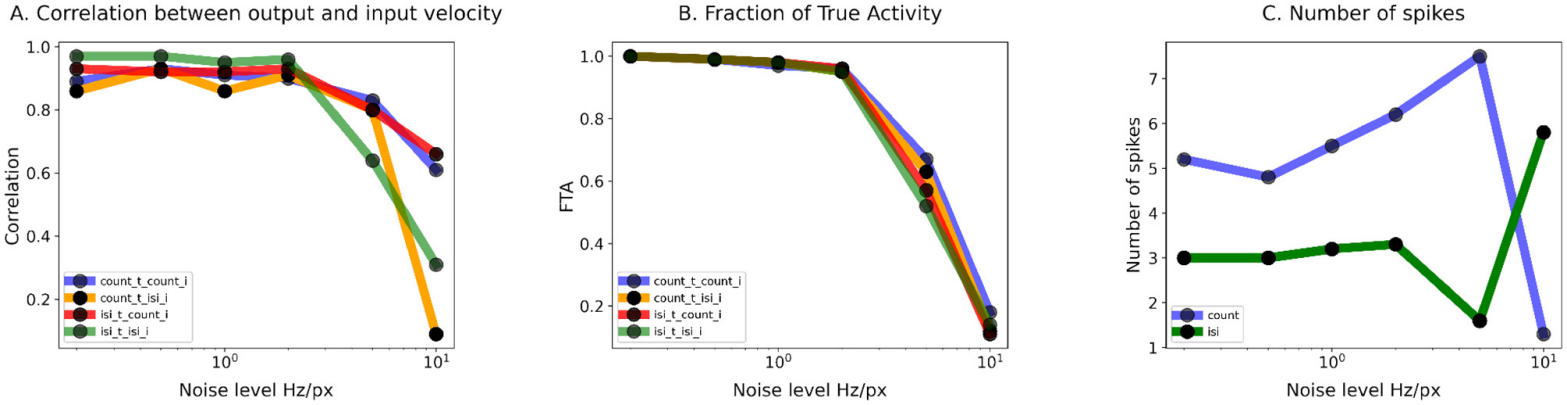
Training of the TDE-3: Summary statistics of TDE performance in the presence of noise. **(A)** Correlation of the TDE output with ground truth input velocity as a function of noise magnitude. **(B)** FTA as a function of noise magnitude. **(C)** Average number of spikes used for encoding of an edge velocity as a function of noise level. In all three cases, noise levels are plotted on the logarithmic scales. For panels A and B the color code is as follows: Blue—training with spike count, inference with spike count during the test; Orange—training with spike count, inference with ISI during the test; Green—training with ISI, inference with ISI during the test; Red—training with ISI, inference with spike count during the test. For **(C)** blue describes training with spike count, green—training with ISI.

**Figure 9:**
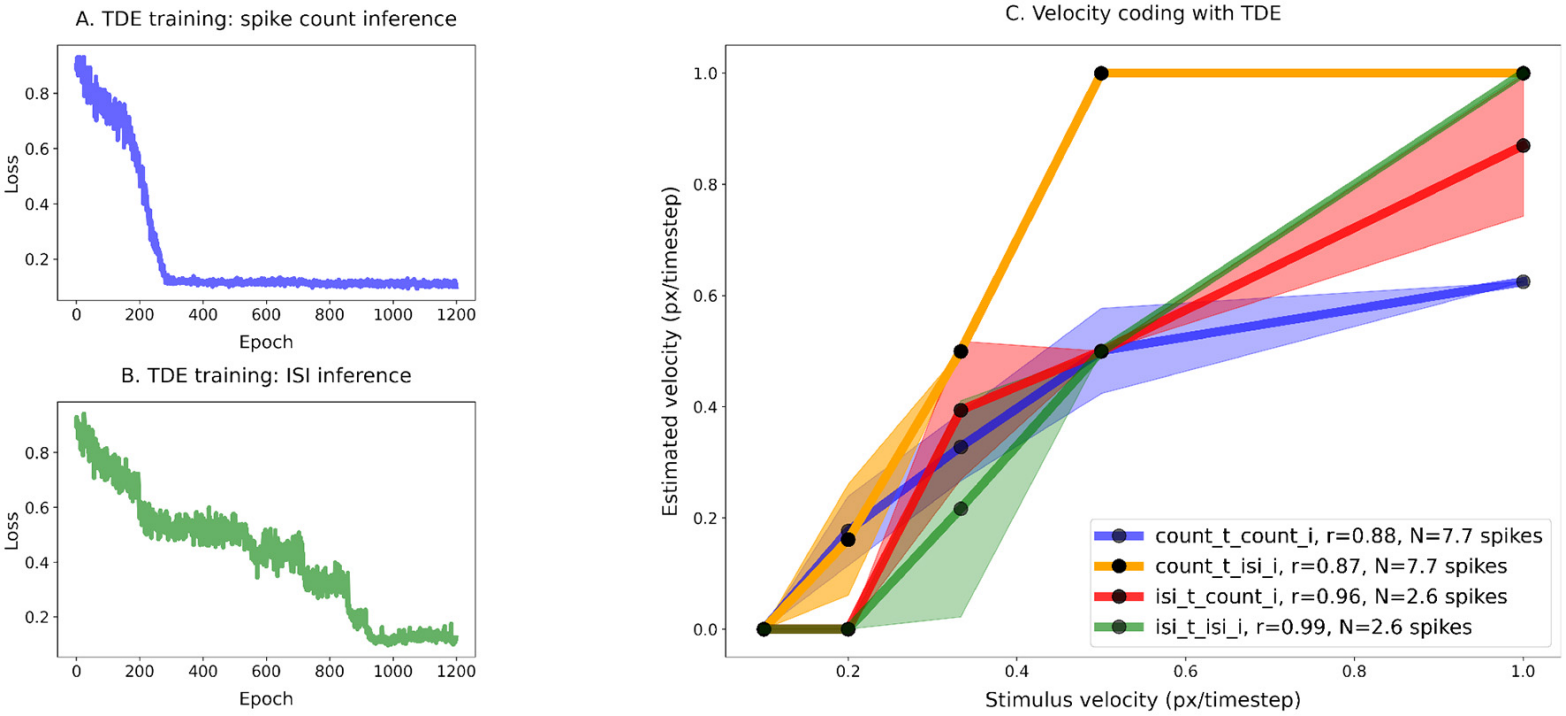
Training of the TDE-3: Robustness to variation in spatial frequency. Wide dynamic range, low resolution, two moving edges at randomly assigned distances. **(A)** Loss function for spike count-based inference, **(B)** Loss function for ISI-based inference. **(C)** Comparison of the velocity tuning curves. Blue - training with spike count, inference with spike count during the test; Orange—training with spike count, inference with ISI during the test; Green—training with ISI, inference with ISI during the test; Red—training with ISI, inference with spike count during the test. As there was no noise, all of the variation in TDE response (shading) originates from the variation in stimulus spatial frequency (spacing between the edges).

**Figure 10:**
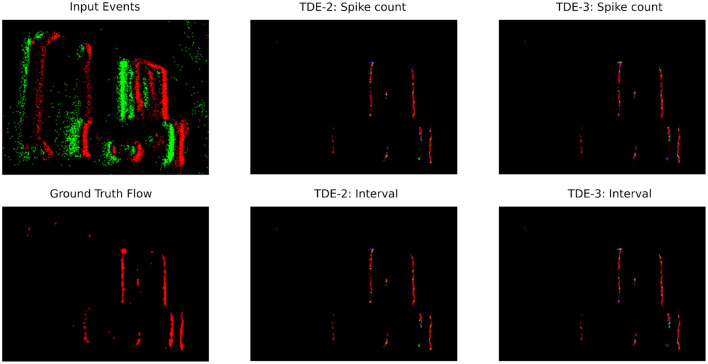
Optical flow coding by TDE: translating boxes. Raw events (top left panel) are color-coded as green-ON events, red—OFF events. Optical flow (the rest of the panels) is color-coded according to Figure 10.

**Figure 11:**
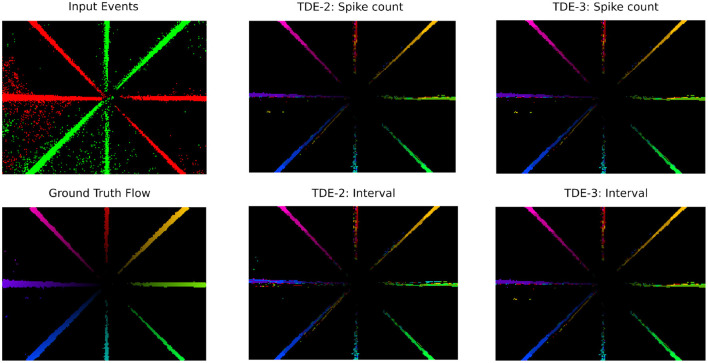
Optical flow coding by TDE: rotating disk.

**Figure 12:**
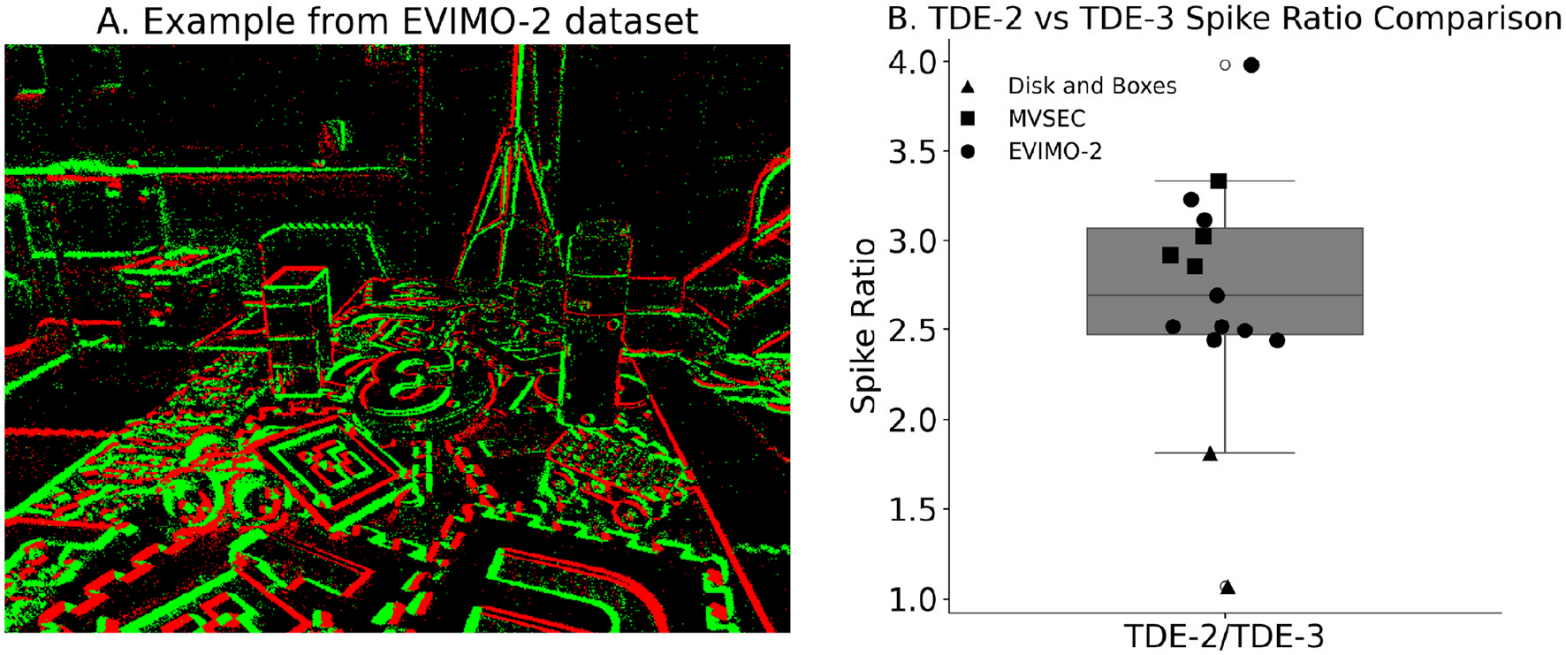
Circuitry energy efficiency. **(A)** Example of the EVIMO-2 dataset. On events—green, Off events—red. **(B)** Ratio of the spikes emitted by TDE-2 and TDE-3 based networks for the three datasets: triangles—disk and boxes from Rueckauer and Delbruck (2016); squares—MVSEC driving sequences; circles—EVIMO-2.

**Figure 13:**
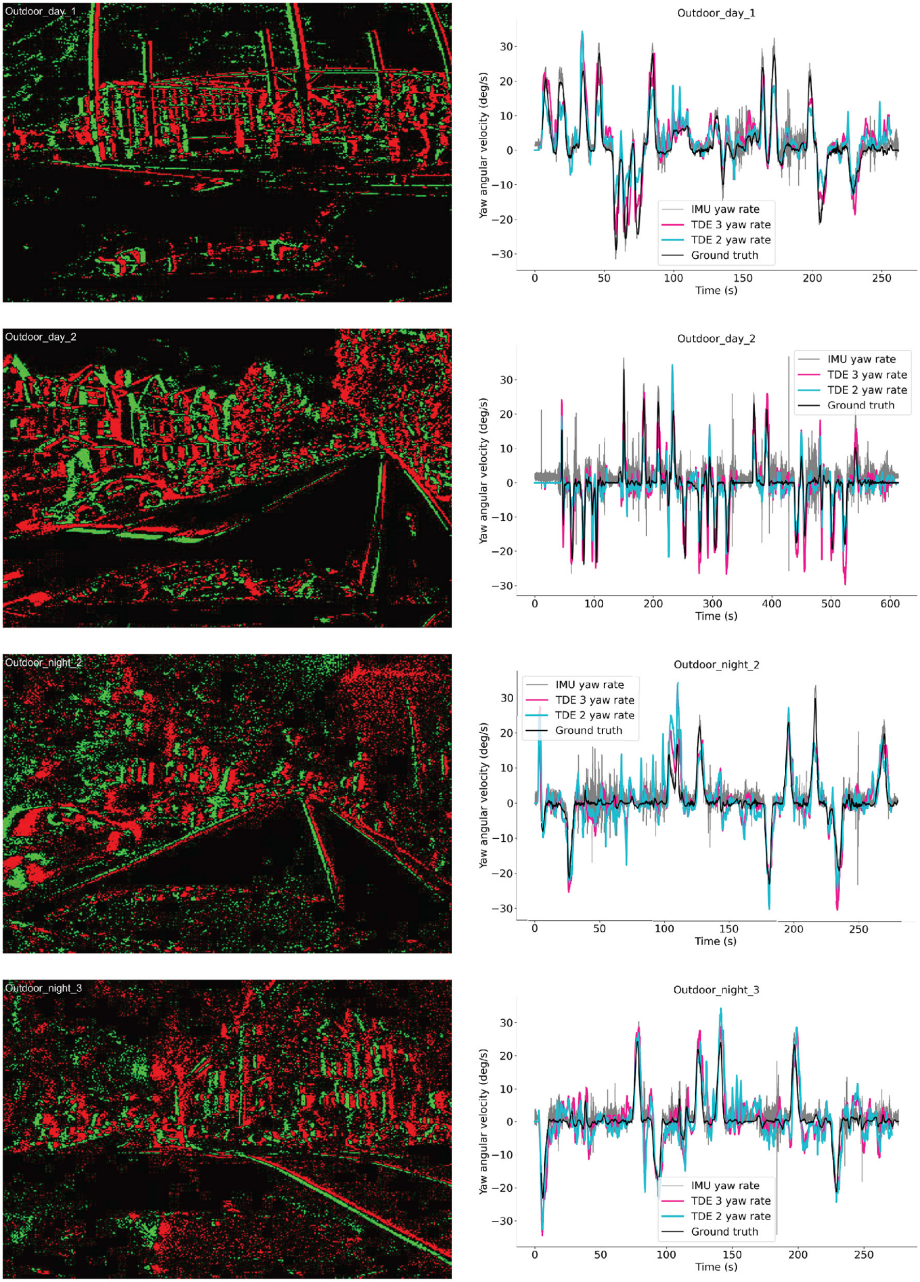
Ego-motion estimation from MVSEC dataset. Left column: examples of the event-based input. Green—On events, Red- Off events. Right column: yaw angular velocities as functions of time for four testing sequences. Black—ground truth angular velocity, Gray—IMU data, Cyan—TDE-2, Deeppink—TDE-3.

**Figure 14:**
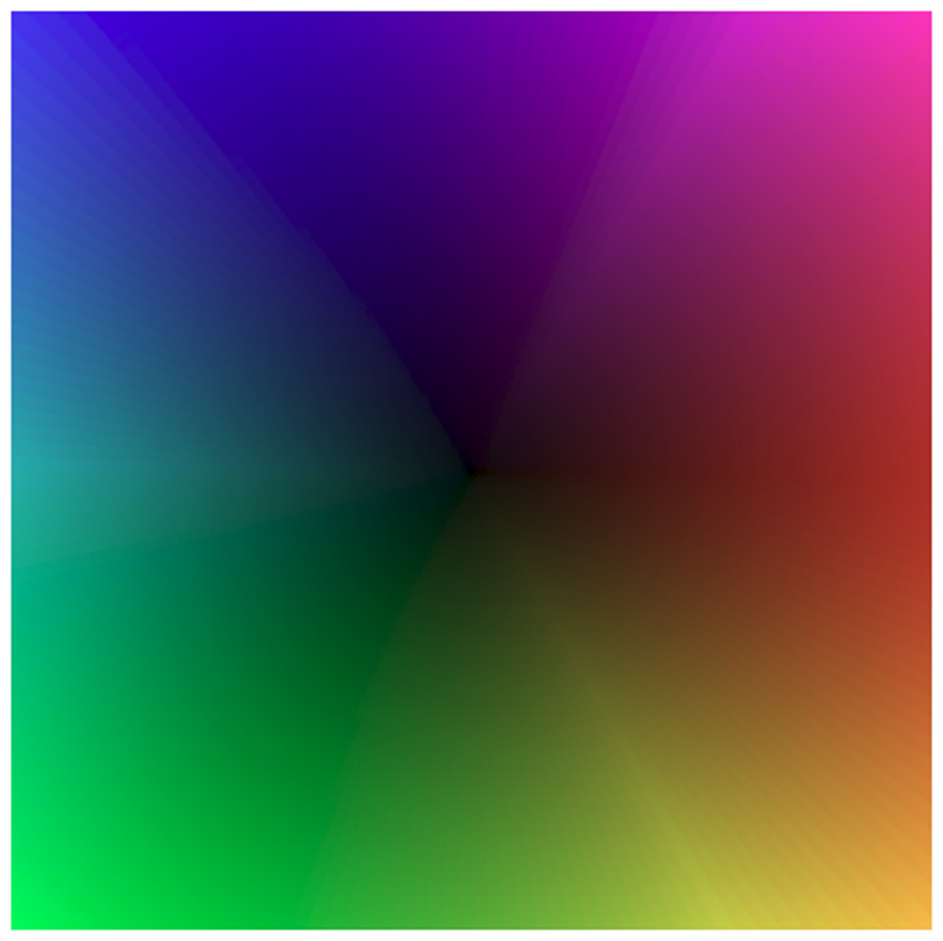
Color map employed for the visualization of the optical flow. Direction is encoded with hue, magnitude with brightness.

**Figure 15:**
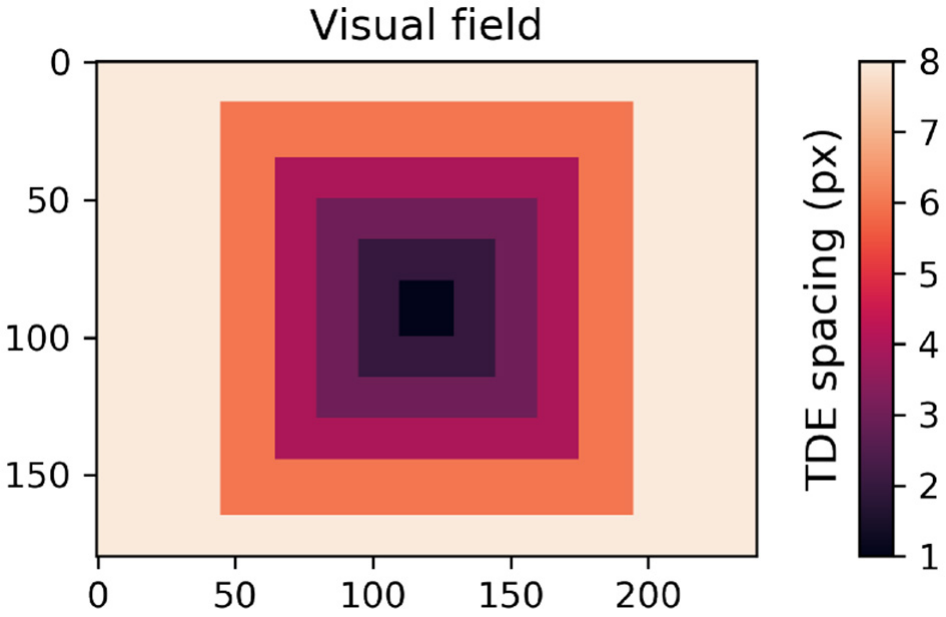
Spacing between compartments of TDE as a function of its location within the visual field.

Upon fixing issues related to detector architecture, we performed a more in-depth study of the TDE coding of velocity with synthetic and real-world data. Specifically, there are five major contributions of this paper:

1. We present more robust TDE-3 and highlight its advantage over the TDE-2 on the level of direction-selectivity of a single detector (Figures 1, 2).
2. We develop a pipeline for the supervised training of the TDE using Back-Propagation Through Time (BPTT) and surrogate gradients (Neftci et al., 2019; Zenke and Vogels, 2021) to linearly map input velocities into an output spike count (Figure 3) or an inter-spike interval (ISI) between the first and the second spike (Figure 4) within both wide (10-fold, Figure 5) and narrow (1.5-fold, Figure 6) dynamic range with precision of up to 2% (Table 1).
3. We develop a novel approach for training SNNs to have a specific ISI using BPTT. While most other methods (Bohte et al., 2002; Mostafa, 2018; Comsa et al., 2020) model the relationship between input magnitude with differentiable function that is then used by BPTT, we measure ISI from the amplitude of low-pass filtered spike train to use BPTT with surrogate gradient (see Section 5.2.2 for the details). The theoretical advantage of our method is that it does not require estimation of the relationship between spike timing and input magnitude, allowing work with more conventional activation functions.
4. We compare the robustness of velocity coding with TDEs to noise (Figures 7, 8) and spatial frequency (Figure 9) composition of the scene—two major challenges for velocity coding with correlation-based motion detectors. Our results show that spike count is more robust to noise, while ISI is less affected by changes in spatial frequency and provides faster velocity inference.
5. We performed quantitative characterization of resolution, robustness, and energy efficiency of encoding real-world optical flow (Figures 10–12, Tables 3, 4) and ego-motion (Figures 13–15, Tables 5, 6) by TDEs. For the optical flow, TDE performs on par with model-based methods of local motion estimation (Rueckauer and Delbruck, 2016; Gallego et al., 2022) (directional error < 20°), but has the advantage of being naturally compatible with spike-based neuromorphic hardware. For the ego-motion, TDEs performed on par (and sometimes even better) with neural networks with 1.5 × 10^5^ parameters (angular velocity error of ≈3°/s). In all of the experiments, TDE-3-based networks were more energy-efficient than TDE-2-based, requiring on average 2.6 × fewer spikes (Figure 12).

**Table 1:** High-resolution velocity inference with the TDE.

**Training inference**	**Test inference**	**Correlation, r**	**Relative error (%)**	**N spikes**
spike count	spike count	0.99	2.5	5.6
	ISI	0.91	30(7.9^*^)	
ISI	spike count	1.0	2.1	7.4
	ISI	1.0	2.7	

^*^Here first two input velocities generated only 1 spike. Therefore, the estimated velocity was 0, which led to a large error in the estimated velocity. We excluded those two values from the analysis to get a better feel of the actual coding error when the velocity can be estimated and get the value of 7.9%.

To summarize, the paper presents a robust computational primitive to optimize the estimation of optical flow from event-based data, compatible with spike-based neuromorphic hardware for low-power inference.

## 2 Time-difference encoders: principles of operation

Before discussing the main findings of the paper, it is important to understand the TDE-2 and TDE-3 computational principles in terms of direction-selectivity and velocity coding to get insight into the features and limitations of the systems under investigation.

### 2.1 Computational primitive and detector architecture

The TDE-2 computational principle is to extract temporal correlations at two spatial locations: facilitator and trigger. A spike input to the facilitator leads to activation of a time-decaying gain, while a spike input to the trigger converts the gain into an excitatory current which charges CuBa-LIF neuron and leads to spiking (Milde et al., 2018) (Figure 1). Essentially, the time-decaying gain extracts temporal correlations as the neuron reports the coincidence of inputs to the facilitator and trigger within a temporal window of the gain decay and scales the current according to the delay between activation of the facilitator and trigger. Hence, faster motion leads to a higher number of spikes with shorter intervals between them and vice versa.

The direction-selectivity of the neuron is ensured by the combination of the two factors. Its activation in the direction anti-parallel to PD, also known as null direction (ND), is prevented since when activation of the trigger precedes the activation of the facilitator, there is no gain to convert to current (Figure 1A center). In the case of the motion in the direction orthogonal to PD (OD), the facilitator and trigger are activated simultaneously. In the hardware, detector firing is prevented by the delay between the processing of the trigger and gain. On the algorithmic level, the delay is realized through the sequence of computational steps: TDE-2 first processes trigger and then gain. Hence, in the case of OD motion, there is also no gain to be converted to the current at the moment of trigger activation.

The crucial deficiency of such a system is that upon motion in OD or ND, there is no gain to be converted to current **only** at the moment of the trigger's activation by the same stimulus. As in such purely excitatory architecture motion in OD and ND leaves a trace of residual gain activity, another stimulus moving in a non-preferred direction will lead to detector activation (Figure 1A). Hence, upon motion in a highly textured environment, individual TDE-2 can lose direction-selectivity.

Our TDE-3 solves the problem of the residual gain by incorporating a third, inhibitory input. Relative to the gain, the inhibitory input is located on the opposite side of the detector and upon activation resets the gain to zero (Figure 1). As a result, when secondary stimuli are moving in the ND, they will first appear at the inhibitory flank. The activation of inhibitory input will remove any residual gain activity, such that when the stimulus activates the trigger, there again will be no gain to be converted to current. In the case of motion in the OD, detector activation will be prevented by simultaneous processing of facilitator and inhibitory inputs (assuming stimuli wide enough to cover 3 inputs) such that the OD motion will leave no trace in gain activity. Thus, the addition of the inhibitory flank allows the detector to retain direction-selectivity in textured environments (Figure 2). As a consequence of such more robust direction-selectivity of individual TDEs, the entire network can spike up to 2–4 times less (Table 6).

### 2.2 Velocity coding with TDE

When the moving stimulus passes the TDE, two properties of the emitted spike train allow downstream circuitry to estimate velocity: the number of spikes and ISI (Milde et al., 2018; Gutierrez-Galan et al., 2022). The spike count is directly proportional to the input velocity, while the ISI is inversely proportional. Fast motion means a short temporal delay between the activation of the gain and its conversion to current. Consequently, the current is large and the number of emitted spikes is high. Conversely, vigorous firing means that spikes closely follow each other and the interval between them is small (Milde et al., 2018). Of special interest is ISI between the first two spikes as it allows very fast velocity inference (Milde et al., 2018).

To decode stimulus velocity from TDE activity in the presence of multiple edges, one needs to perform motion segmentation to relate counted spikes/measured ISIs to a particular moving edge. To this end, we propose to count spikes starting from each activation of TDE's trigger as it indicates passage of a new edge. Additionally, we limited the time window over which spikes are counted for spike count inference. Otherwise, in the presence of multiple edges motion of the very last edge would affect the measured velocity of the very first edge.

From the perspective of a single neuron's coding capacity, and regardless of the inference method, all-or-none spiking is more of a liability than an asset. All-or-none spiking is a liability, because it introduces a latency vs. dynamic range dilemma, as spiking occurs in time (Rieke et al., 1999). For example, to discriminate input velocities within a range of, e.g. 100 different values, would require a coding window of at least 100 timesteps either to count spikes, or to measure interspike intervals. Even with a very short timestep of 1 ms, the lag becomes substantial for edge applications like robotics, where neuromorphic hardware is needed the most and low latency is paramount. Thus, to avoid impractical latency, one can either encode a narrow range of input velocities with high resolution, or a wide range of input velocities with low resolution (Figures 5, 6). Therefore, a strategy to reconcile latency and dynamic range requirements would be to use each detector within a relatively narrow range of velocities, while boosting the dynamic range of the entire network by using detectors with different spacing between detector inputs.

The coding of velocity by the TDE is additionally troubled by the noise and spatial frequency of a scene. Noise leads to the non-stimulus-related activation of detectors. Spatial frequency biases detector responses as edges closely following each other will lead to stronger TDE activation than a single edge. In the results section, we compare spike count and ISI-based inference in terms of their robustness in various noise (Figures 7, 8) and spatial frequency (Figure 9) conditions. We observe that spike count is more robust to noise, while ISI is less biased by scene spatial frequency.

## 3 Experiments and results

The experiments that we performed can be classified into three categories: (1) investigation of the robustness of the direction-selectivity of individual TDE-3 and TDE-2 (Figure 2), (2) training TDEs to linearly map input velocities into output (Figures 5–7), and (3) application of the TDEs for optical flow and ego-motion inference from real-world data (Figures 10–12).

### 3.1 Robust direction-selectivity of individual TDE-3 in textured environments

To compare the direction-selectivity of TDE-2 and TDE-3, we simulated their responses to textures moving in four cardinal directions (Figure 2B): left-to-right (L-R), right-to-left (R-L), top-to-bottom (T-B) and bottom-to-top (B-T). For each stimulus presentation (2000 per testing round), we randomly varied the texture composition, motion direction, and velocity (see Section 5.1 for the details). To show that the robust direction-selectivity of the TDE-3 stems from its structure and is not due to some particular set of parameters, we varied at each testing round (400 in total) all of the TDE parameters over a 10-fold range. Without loss of the generalization, we limited our investigation of direction-selectivity to responses of L-R motion-sensitive TDEs (Figure 2B) that sampled signals from three adjacent pixels through which textures were moving.

Figure 2B shows how the responses of TDE-2s and TDE-3s depend on the texture's direction of motion. One can appreciate that while L-R TDE-2 fires to all stimuli directions, the spiking of the TDE-3 is contained to stimuli in its preferred direction. To assess the TDE's direction-selectivity quantitatively, we calculated the so-called Direction-Selectivity Index (DSI) as the ratio between the number of spikes emitted by motion in PD and the total number of spikes fired by the TDE in a testing round. Figure 2C compares the DSI of TDE-2s and TDE-3s averaged across all of the testing rounds. For the TDE-2, the mean DSI is 0.36 with a standard deviation of 0.08. Such DSI indicates a rather poor direction-selectivity in a textured environment, because on average inference of motion direction with such a detector is only 11% better than random guessing. On the other hand, the TDE-3 has a DSI of exactly 1, with zero deviation. DSI equal to 1 means that regardless of the initialization of the parameters, simply because of its structure with inhibitory input that eliminates residual gain activity (details—Section 2, illustration—Figure 1), the TDE-3 is activated only by motion in the PD.

For simplicity of interpretation, in Figure 2 we limited our analysis to 1D motion. Yet, the same conclusion holds for the 2D motion as well (Supplementary Figure 1). We performed experiments with textures of increasing complexity (vertical bars, checkerboard, randomly generated textures) moving in 2D and showed that in all of the cases, TDE-3 had higher DSI than TDE-2. However, an increase in texture complexity decreased the DSI of TDE-3 (see Supplementary Figure 1 for details).

### 3.2 Supervised training of TDE-3

To promote linear mapping between the input velocities in PD and the TDE outputs, we performed supervised training with BPTT and surrogate gradients for both spike count and ISI-based inference methods and compared obtained results (see Section 2 and 5.2 for the details). Note that in the context of linear mapping of input velocities into TDE outputs, the TDE-2 and TDE-3 are identical because the delay between the activation of the facilitator and trigger is not affected by the inhibitory input. Therefore, although in experiments with synthetic data we perform all of the training on the TDE-3, the results are generalizable to the TDE-2.

There were three types of experiments with training of TDE-3. First, we established that detectors are trainable in principle and that linear mapping between input velocities in a given cardinal direction and TDE output is possible for various stimuli dynamic ranges (Figures 5, 6, Table 1). Second, we investigated how training and mapping are affected by changes in the spatial frequency of the stimulus—a known liability of correlation-based motion detectors (Borst and Euler, 2011) (Figure 9). Third, we studied the robustness of TDE training and inference in the presence of various levels of noise (Figures 7, 8).

#### 3.2.1 Linear mapping of velocity of moving edge

To study how the TDE can map the input velocity into its output, we simulated the TDE responses to a single edge moving from left to right at various constant velocities. We opted for these stimuli because they directly target the goal of linear mapping while keeping things simple and unambiguous. To avoid overfitting and the necessity to separate stimuli into validation and test datasets, we randomly assigned the velocity of an edge at each stimulus example presentation. We employed two stimuli sets. One contained 5 velocities within a 10-fold range of magnitude and was used to show that the TDE can encode velocities in a wide dynamic range. The second stimuli set contained 15 different velocities spanning 1.5-fold range of magnitudes and was used to show that the TDE can encode velocities with fine resolution (see Section 5.1).

As a metric of the quality of linear mapping, we used the Pearson correlation coefficient between the stimulus and the estimated velocity (Pearson, 1895). The performance of the trained TDEs was assessed with both ISI and spike count regardless of which one was used during training to study the connection between these two inference methods. Additionally, to assess the dynamic energy efficiency of the trained detectors, we calculated the average number of spikes elicited by a stimulus.

Figure 5 shows that the TDE can be trained to encode a wide dynamic range of input velocities. The loss function on panels A (training with the spike count) and B (training with ISI) gradually decreases during training, indicating that with both inference methods it is indeed possible to train the TDE to discriminate input velocities in a wide dynamic range.

Figure 5C makes a quantitative comparison of velocity coding with various training and inference methods. Unsurprisingly, the correlation coefficient between estimated and true velocity is the highest when evaluation is done with the same inference method as training: r = 0.97 for the spike count training and inference (blue line), r = 0.99 for the ISI training and inference (green line). Yet, training with spike count leads to reasonable performance with ISI inference (r = 0.86, orange line) and vice versa (r = 0.85, red line), with the only problem being distinguishing between the two highest velocities. Training with ISI-based inference also leads to a lower average number of spikes emitted by stimulus (1.9 vs. 4.4), suggesting higher energy efficiency when implemented on neuromorphic hardware (Caccavella et al., 2023).

Figure 6 demonstrates that the TDE can effectively encode a narrow dynamic range of input velocities with high precision (Panels A and B). In Panel C, one can appreciate that apart from the case of training with spike count and evaluation with ISI (orange line, *r* = 0.91), input velocities are mapped into TDE outputs nearly perfectly, with a correlation *r*≈1.0 (Table 1). Also, ISI evaluation with spike count training results in a decrease in dynamic range, as the estimate becomes zero for the first two velocities. This occurs because these two almost equal velocities (0.0256 and 0.0263) were encoded with 1 spike, making the ISI indeterminable.

For this experiment, fine resolution was paramount. Therefore, we calculated mean relative error by dividing the difference between true and inferred velocity by true velocity and averaging this metric across all velocities (see Equation 5, Table 1). Unsurprisingly, ISI evaluation with spike count training yielded the largest relative error (30%). Even when excluding the first two velocities, which, because TDE emitted only 1 spike, contributed to the variance the most, the relative error remains >= 3 times higher (7.9%) than for the other three conditions, where velocity was encoded with an error as small as 2.1% (ISI training, spike count evaluation, red line). We also note in this experiment that training with ISI led to a higher average number of spikes (7.4) compared to spike count training (5.6) (see Table 1).

To conclude, the TDE can be trained to successfully encode the velocities in wide and narrow ranges.

#### 3.2.2 Spatial frequency and velocity coding by TDE

When multiple moving edges are present, temporal signal integration by current and voltage compartments could lead to biases in velocity estimation due to variations in distance between moving edges (i.e. spatial frequency). To test whether training of the TDE-3 can mitigate this problem, we trained TDE-3 to linearly map the velocities of two moving edges into the spike count and ISI while varying the distance between edges in a 10-fold range (see Section 5.1). W employed a wide dynamic range of velocities (see Section 5.1) as it presents a more natural kind of stimuli due to optical flow in real-world scenes varying over a large range of apparent velocities (Mitrokhin et al., 2019b; Rueckauer and Delbruck, 2016).

Figure 9 shows that one can train the TDE to linearly encode velocities of multiple moving edges with both spike count- (Panel A) and ISI-based (Panel B) inference. In Panel C, we plotted the obtained tuning curves of the responses to the second edge for all 4 testing combinations. Shading indicates standard deviation in response to a given velocity and originates from the variation in distance between edges. Panel C shows that regardless of inference during testing, training with ISI (ISI test -green line, r = 0.99; spike count test -red line, r = 0.96) leads to a higher correlation with stimulus velocity than training with spike count-based inference (spike count test— blue line, r = 0.88; ISI test—orange line, r = 0.87). However, training with ISI-based inference tends to constrict dynamic range, effectively encoding velocities over a 3-fold range. Training with spike count-based inference preserved almost the entire dynamic range of input velocities but saturated at the highest velocities. Training with spike count also led to a higher average number of emitted spikes 7.7 vs 2.6. Noteworthy, regardless of the inference method employed during training, spike count-based inference during testing resulted in higher variation in the estimation of velocity.

To conclude, Figure 9 shows that the trained TDE is fairly robust to variation in stimulus spatial frequency.

#### 3.2.3 Noise and velocity coding by TDE

To test whether training of the TDE to encode velocity is robust to noise, we injected background activity noise into our simulation–one of the primary sources of noise in event-based vision (Guo and Delbruck, 2023). Noise was modeled as stochastic events following a Poisson distribution, with mean rates varying over a 50-fold range: from 0.2 Hz/px to 10 Hz/px (see Section 5.1). The same noise levels were applied during training and during testing.

To allow the network to combat noise, we employed a Spatio-Temporal Correlation Filter (STCF) of events (Section 5). STCF is a popular noise-combating tool in the field of event-based vision (Guo and Delbruck, 2023; Rueckauer and Delbruck, 2016; Gallego et al., 2022) that allows an event to pass the filtering stage only when there are events in at least n neighboring pixels within a specified time window (Section 5.2, Guo and Delbruck, 2023), with n being a trainable parameter. Although, strictly speaking, as a result we would test not the performance of the TDE, but of the TDE and STCF, our approach is still sound. The reason for this is that STCF is almost always part of the TDE circuitry (Milde et al., 2018; Schoepe et al., 2024; D'Angelo et al., 2020; Gutierrez-Galan et al., 2022; Chiavazza et al., 2023). Therefore, since in real applications TDE will always be paired with STCF, it makes sense to investigate their joint performance. Especially, since STCF does not eliminate all of the noise and there is always a concern that aggressive filtering would destroy part of the signal.

In the presence of noise, not every increase in current is caused by a moving edge. Therefore, one of the goals of the training is to suppress the TDE activations caused by noise. To account for this factor in the loss function, we calculated the loss between all of the velocity estimates performed by the TDE and stimulus ground truth at the corresponding timepoints (effectively, this ground truth was zero everywhere except at the moment of edge appearance). Also, to evaluate how the trained TDE can reject noise and preserve true motion signal, we employed an additional metric called fraction of true activity (FTA) as the sum of estimated velocities caused by the stimulus (true motion signal) to the total sum of estimated velocities (caused by signal and noise).

Figure 7 shows the results of the training for two extreme cases of “bright” (noise rate=0.2Hz/px) and “dark” (noise rate=5Hz/px) scenes. With low noise level, the performance of the TDE (Figure 7 top row) was almost identical to the performance in noiseless experiments (Figure 9) in terms of the shapes of the tuning curves and the correlation with stimulus velocities. These observations are also confirmed by FTA (Figure 8) with almost all TDE activity being caused by the stimulus. The results of the training with a high level of simulated noise are shown in Figure 7, bottom row. With both inference methods, there is an early drop in the loss function (AII and BII panels) caused by an increase in the number of neighboring pixels where at least one event should occur for any given event to pass the STCF processing stage (see Section 5.2). As a result of this, more noise is rejected and loss dramatically decreases.

Panel CII illustrates tuning curves obtained in all 4 testing conditions. One can immediately note that training and inference with ISI (green line) leads to massive standard deviation (shading) in the estimated velocity. Surprisingly, TDEs trained with ISI showed better performance when testing was done with spike count (red line) as the standard deviation was lower and the correlation coefficient was higher (r = 0.8 vs. r = 0.64). With ISI-based training, about half of the encoded motion signals were related to the stimulus (Figure 8). Yet, given the low mean spike count (*N* = 1.6), TDEs coding capabilities were rather poor, with at least one edge being ignored most of the time. Training with spike count-based inference leads to much better performance with both spike-count (blue line and shading, r = 0.83) and ISI-based (orange line and shading, r = 0.8) evaluation. Spike count-based training leads to a mean spike count of 7.5 spikes. Since with this training condition almost 70% of encoded motion signals were related to the stimulus (Figure 8), we conclude that spike count-based training allows TDE to fairly reliably encode motion signals in the presence of noise.

In Figure 8 we summarized the influence of the noise levels on the correlation between output and ground truth input velocity (panel A), FTA (panel B), and mean number of spikes generated by a moving edge (panel C). Panels A and B show that TDE-3 is very robust up to a noise level of up to 2Hz/px with FTA >95% and correlation with the stimulus velocity r of ≈ 90% throughout and largely regardless of the training / inference method. However, a further increase in noise level led to a sharp decrease in performance with most of the TDE output being noise-driven. Our results also show that in the presence of noise, the spike-count-based inference serves as a more robust and reliable training signal. The likely reason for this is that ISI dependency on only two spikes makes it susceptible to noise, whereas spike count measures signals over a certain time window, and therefore mitigates variation in response caused by exact spike timing.

### 3.3 Real-world data

We studied the performance of the TDE-2 and TDE-3 with real-world data on the levels of (i) estimation of the local motion, (ii) estimation of the global motion (ego-motion), and iii) energy efficiency. In all of the experiments, the real-world data was used as a test dataset. TDEs were pre-trained on synthetic data (see Section 5.2.3) using spike count as the inference method because it gives better performance in the presence of noise (Figure 8). We pooled ON and OFF events as often encountered in biological systems (Clark and Demb, 2016; Borst et al., 2020; Sterling and Laughlin, 2015). The neuronal parameters used in these experiments are listed in the Table 2.

**Table 2:** Ego-motion network parameters.

**Parameter**	**Value**
Timestep duration (*dt*)	50 ms
Synaptic weight	2.37
Time constant, gain	252 ms
Time constant, current	470 ms
Time constant, membrane	153 ms
Time constant, *A*	750 ms
STCF, spatial neighborhood	6
STCF, temporal window	50 ms

#### 3.3.1 Local optical flow estimation

To study coding of the local optical flow, we employed sequences of boxes moving to the right and counterclockwise rotating disk from the dataset presented in Rueckauer and Delbruck (2016). The former is an example of textured stimuli with multiple edges moving in the same direction and thus directly tests the robustness of TDE direction-selectivity and gains obtained by augmenting the TDE-2 with an inhibitory input. The latter contains a high dynamic range of velocities in both magnitude (80-fold) and direction (360°) and is used to probe velocity coding by TDEs in real-world scenes.

##### 3.3.1.1 Translating boxes

We start the analysis of TDE responses to boxes moving to the right (Figure 10) by estimating the direction-selectivity of individual TDEs in a manner similar to DSI (Figure 2). Although strictly speaking, here we cannot calculate the DSI because the motion of the boxes was confined to one direction, we can approximate it by calculating the fraction of total spikes in the network that belonged to the detectors tuned in the L-R direction. Ideally, this number should be one, as only L-R detectors should spike. Table 3 shows that for the network of TDE-2 only 43% of spikes are from the L-R detectors, while for the network of TDE-3 70% of spikes are attributed to L-R detectors. Thus, just as in the case of synthetic data (Figure 2), the TDE-3 has more robust direction-selectivity.

**Table 3:** TDE performance with real-world data: translating boxes.

**Detector type**	**Inference method**	**mean AAE ± std (°)**	**Fraction of spikes in PD**	**Total N spikes**
TDE-2	Spike count	19 ± 31	0.43	229686
	ISI	18 ± 31		
TDE-3	Spike count	18 ± 32	0.7	126692
	ISI	17± 31		

TDEs were trained with spike count inference (see Section 5.2.3).

Next, in Figure 10 we visualized optical flow coding by the TDEs using the colormap from Figure 14. Surprisingly, although the TDE-3 has a more robust direction-selectivity on the level of individual detectors, on the network level the TDE-2 and TDE-3 seem to have similar qualitative performance regardless of inference methods. This qualitative observation is also confirmed quantitatively: Table 3 indicates that in all of the testing conditions, the Average Angular Error (AAE) (≈ 20°) and standard deviation of the angular error (≈ 30°) were almost identical.

The apparent contradiction between responses at the level of individual TDEs and at the network level arises because the estimation and visualization of optical flow require subtracting the outputs of detectors tuned to opposite motion directions (Section 5.2.3). When an edge is moving to the right it generates residual gain activity in T-B, B-T and R-L tuned detectors which would be converted to TDE output upon appearance of a second edge moving to the right (Figures 1, 2, Section 2). For B-T and T-B tuned detectors, this activation should be equal (except for noise), whereas activation of L-R tuned detector would be always higher than activation of R-L tuned detectors. Consequently, the subtraction of velocities estimated by the opposingly tuned detectors compensates for poor direction-selectivity of individual TDE-2s by removing signals caused by residual gain activity from the data stream.

To conclude, networks of TDE-2s and TDE-3s can both reliably infer motion direction in natural scenes with spike count and ISI-based inference.

##### 3.3.1.2 Rotating disk

The ability of TDE to encode motion in a wide dynamic range of direction and magnitude was tested with the aid of a rotating disk sequence (Rueckauer and Delbruck, 2016) (Figure 11 top panel) that challenged networks of TDE with 360° range of motion direction and 80-fold range of velocity magnitude. To deal with such stimuli conditions, the network of TDEs tuned to 4 cardinal directions sampled the space anisotropically, with the lower distance between the TDE inputs in the center and higher in the periphery (Figure 15). This bioinspired approach (D'Angelo et al., 2020) allowed us to reconcile the dynamic range, resolution, and latency requirements (see Section 2, 5.2). We evaluated optical flow coding by networks of TDE-2 and TDE-3 with spike count and ISI-based inference.

Figure 11 employs a color code scheme from Figure 14 to visualize the optical flow coding of various TDE networks to compare with ground truth (Figure 11 left bottom). The figure indicates that in all 4 testing conditions, there is a close correspondence between TDE inference and ground truth. This observation is also supported quantitatively. In all four cases, TDE showed similar [and fairly low, see Rueckauer and Delbruck (2016)] AAE (mean and std of ≈ 20°, Table 4) and relative average endpoint error (rAEE, mean of ≈ 0.4, std of ≈ 0.2).

**Table 4:** TDE performance with real-world data: rotating disk.

**Detector type**	**Inference method**	**mean AAE ± std (°)**	**rAEE ± std**	**Correlation, r**	**Total N spikes**
TDE-2	Spike count	21 ± 24	0.43 ±0.23	0.87	1638878
	ISI	21 ± 24	0.47 ± 0.18	0.88	
TDE-3	Spike count	22 ± 24	0.45 ± 0.26	0.87	1532557
	ISI	21 ± 24	0.47 ± 0.2	0.88	

Although an error of 40% in the estimation of velocity might seem substantial in terms of coding precision, it is important to keep three things in mind. First, we encode over a wide dynamic range of velocities. Secondly, each detector encodes velocities with few spikes/short ISI, so, naturally, such coarse binning would impair precision. However, most importantly, there is an almost 90% correlation between the estimate of the TDE velocity and ground truth (Table 4). The high correlation with ground truth is the most important result, because since optical flow depends on the ratio between velocity and distance, it does not provide information about absolute velocities anyway (Serres and Ruffier, 2017). Meanwhile, a high correlation with velocity indicates that regardless of the exact scale, networks of TDE can reliably determine the relative distribution of the velocities in the visual scene.

To summarize, both TDE-2 and TDE-3 can extract a wide dynamic range of velocity direction in magnitude from real-world event-based data with both spike count and ISI-based velocity inference.

#### 3.3.2 Ego-motion estimation

To assess how well networks of TDE-2/TDE-3 can estimate global optical flow caused by ego-motion we used 4 outdoor driving sequences (2 day-time, 2 night-time, Figure 13 left column) from the MVSEC dataset. We followed the approach of Greatorex et al. (2025) where the yaw rotation rate (angular velocity) is estimated by integrating spikes from L-R detectors with a positive sign and spikes from R-L detectors with a negative sign. The spacing between TDE inputs was 2 (Greatorex et al., 2025).

Now, since we integrate over the space, theoretically the estimates of the angular velocity are biased by amount of texture in the scene: multiple slowly moving objects would lead to larger spike count than few quickly moving ones. However, in practice it is less of a problem because large turns (high angular velocity) usually occur on the crossings, and in cities crossings tend to have quite a lot of texture.

Still, there remains the problem of scale: how should one convert into °/s? Here, in our proof-of-principle study, we simply normalized output such that maximal activity corresponds to maximal angular velocity across all sequences. Since peak angular velocity depends only on dynamics of a vehicle, it is reasonable to assume that we know what our peak activity would correspond to. However, we know what was the peak activity only after the sequence played out, while ideally one would like to estimate it in real-time. In Figure 13 (right column) we plotted the estimated angular velocities with TDE-2 (cyan trace), TDE-3 (deeppink trace), together with the ground truth (black trace) and the yaw rotation measurements provided by IMU (gray trace) for the 4 aforementioned outdoor motion sequences. The IMU serves a nice benchmark estimating yaw rotation rate using standard off the shelf sensor that operates in non-visual domain to which no postprocessing was applied. The plots show that all three ways to estimate angular velocity reasonably follow ground truth yaw angular velocity.

To quantitatively assess performance of the TDE-based networks we calculated errors in the angular velocity estimation (Angular Velocity Error, AVE) and correlation with the ground truth angular velocity for TDE-based networks and IMU. We also calculated number of spikes emitted by TDE-2 and TDE-3 based networks (Table 5). To compare our results with other approaches to ego-motion estimation from MVSEC event-based visual input (Greatorex et al., 2025; Zhu et al., 2019; Mitrokhin et al., 2019a; Ye et al., 2020) we also estimated Average Relative Rotation Error (ARRE, see Methods for the details, Table 6).

**Table 5:** Performance comparison on MVSEC sequences.

**MVSEC sequence**	**Detector type**	**mean AVE ± std (°/s)**	**Correlation, *r***	**Total N spikes**
Outdoor_day_1	TDE-2	3.7 ± 3.5	0.84	1.4 × 10^8^
	TDE-3	3.8 ± 3.0	0.87	4.9 × 10^7^
	IMU	1.9 ± 1.4	0.98	–
Outdoor_day_2	TDE-2	3.2 ± 4.1	0.63	7.0 × 10^8^
	TDE-3	3.1 ± 4.2	0.79	2.1 × 10^8^
	IMU	2.2 ± 2.0	0.93	–
Outdoor_night_2	TDE-2	3.4 ± 3.4	0.80	1.4 × 10^8^
	TDE-3	3.0 ± 2.9	0.85	4.8 × 10^7^
	IMU	1.7 ± 1.5	0.95	–
Outdoor_night_3	TDE-2	3.7 ± 3.3	0.84	1.3 × 10^8^
	TDE-3	3.6 ± 3.1	0.86	4.3 × 10^7^
	IMU	1.6 ± 1.5	0.96	–

Angular Velocity Error (AVE) reported as mean ± std.

**Table 6:** ARRE comparison on MVSEC sequences (rad).

**Method**	**Outdoor_day1**	**Outdoor_day2**	**Outdoor_night2**	**Outdoor_night3**
This work TDE-2	0.00325	0.0028	0.003	0.0032
This work TDE-3	0.033	0.0027	0.0026	0.00315
IMU	0.00166	0.00192	0.00148	0.0014
Mitrokhin et al. (2019a)	0.0994	0.108	0.116	0.121
Ye et al. (2020) [SfM learner from Zhou et al. (2017)]	0.00916	-	0.00499	0.00482
Ye et al. (2020)	0.00267	-	0.00202	0.00202
Zhu et al. (2019)	0.00867	-	-	-
TDE-2 in Greatorex et al. (2025)	0.00065	-	0.00053	0.00067

The same as in the case of moving boxes (Figure 10, Table 3) and rotating disk (Figure 11, Table 4), TDE-2 and TDE-3 had similar mean AVE of ≈3 deg/s, which was almost two times higher than the mean AVE of the IMU. In terms of correlation with the ground truth, TDE-3 had *r* in the range of 0.79 (Outdoor_day_2) to 0.87 (Outdoor_day_1), which is slightly higher than for TDE-2 (0.63–0.84). Yet both of them are considerably worse than the IMU, whose correlation with the ground truth is >95%.

Since most of the other studies that were estimating ego-motion from MVSEC driving sequences were done with neural networks trained to estimate change in orientation between two timesteps, for a proper comparison we also employed ARRE metric, which describes how much the estimated change in orientation differs from the ground truth (Table 6). To obtain change in the orientation we integrated estimated angular velocities over 50ms, that corresponded to the frame rate at which ground truth was available in Zhu et al. (2018a,b).

The results show that our algorithms based on TDE-2 / TDE-3 perform on par with neuronal networks containing 1.5 × 10^5^ trainable parameters (Mitrokhin et al., 2019a). Caution, however, should be applied in interpreting these results. In most of the other approaches (Zhu et al., 2019; Mitrokhin et al., 2019a; Ye et al., 2020) networks estimated pitch, roll, and yaw angles, whereas we estimate only the yaw angle. Although driving cars indeed mainly rotate along the yaw axis, ground truth data show small [≈10 times smaller than yaw rotations Zhu et al. (2018a)] but non-zero pitch and roll angular velocities. Furthermore, in our study, we recovered the absolute scale by normalization, while (Zhu et al., 2019; Mitrokhin et al., 2019a; Ye et al., 2020) had to learn it from the data.

Noteworthy, in Greatorex et al. (2025), where yaw rotations were estimated from the output of TDE-2 in a way similar to ours, the ARRE was at least two times lower than even the IMU. Perhaps the discrepancy between our results and those from Greatorex et al. (2025) can be explained by the presumably higher frame rate at which they processed incoming events. Although we were unable to run our network at such a high frame rate for the entire image, we can replicate the results of Figure 6 in Greatorex et al. (2025), where in total 200 TDE-2 units were used to estimate the angular velocity of the first 90 s in the *Outdoor_day_1* dataset. We ran the network at 10 kHz, but still performed worse than (Greatorex et al., 2025) (Supplementary Figure 2).

#### 3.3.3 Circuit energy-efficiency

Describned above experiments with the real-world data show that TDE-2 and TDE-3 give almost identical performance in terms of encoding of optical flow. The reason is simple: subtraction of the responses of opposingly-tuned detectors removes any residual activity from TDE-2 responses.

Yet, a quick inspection of the column “Total N spikes” in Tables 3–5 shows that TDE-2-based circuitry always spiked more than TDE-3-based circuitry. The reason for this is that although subtraction of velocity estimates made by opposingly tuned detectors mitigates the loss of direction-selectivity of TDE-2 in a textured real-world environment, the inhibitory input to TDE-3 eliminates residual activity immediately at the level of motion detection.

To make a statistical argument for the higher energy efficiency of TDE-3 we compared detector spike count with additional 9 real-world sequences from Burner et al. (2022), example of such a sequence is shown in Figure 12A.

The box plot in Figure 12B shows that, in all three stimulus sets, TDE-2 emitted more spikes than TDE-3. For statistical comparison, we employed linear mixed-effect models, treating dataset type as a fixed (Bates et al., 2015), since sequences from the same dataset are more alike than those from different datasets (because they were recorded with the same hardware and under similar conditions). The analysis showed that TDE-2 emitted significantly more spikes than TDE-3 (mean TDE-2/TDE-3 ratio: 2.7, *p*≈6 × 10^−8^). Thus, although pooled responses of TDE-2- and TDE-3-based networks exhibit the same precision in optical flow estimation, TDE-3-based networks are on average about 2.7 times more efficient in terms of emitted spikes.

## 4 Discussion

In the present study we performed a thorough investigation of the TDE as a computational prior for optical flow estimation in SNNs on three different levels: detector architecture, velocity inference, and real-world performance. Although our study was focused on the algorithmic level, there is little doubt that TDE-3 can be implemented in hardware. First, inhibitory inputs in neuromorphic motion detectors have a long, 30-year history (Kramer, 1996; Deutschmann et al., 1997; Higgins et al., 1999; ROS et al., 1999; Giulioni et al., 2016) and can be readily implemented as a simple switch or logical gate. Second, TDE-3 is a natural extension of TDE-2 and hence is naturally compatible with all of the neuromorphic hardware on which TDE-2 was implemented: from custom-manufactured chips to Loihi 2 (Milde et al., 2018; Gutierrez-Galan et al., 2022; D'Angelo et al., 2020; Chiavazza et al., 2023; Schoepe et al., 2024; Greatorex et al., 2025).

Below, we summarize the key outcomes of the study and discuss possible future improvements.

### 4.1 Detector architecture

On the level of detector architecture, we showed that classical TDE-2 loses direction selectivity in a textured environment due to residual activity in the gain compartment (Figures 1, 2, 10). We proposed a bio-inspired solution for this problem: augmenting the TDE-2 with an inhibitory input that resets the gain and removes residual activity upon activation (Figures 1, 2, 10). This procedure preserves the direction selectivity of individual TDE-3s (Figure 2, Table 3) and increases the computational efficiency of the network of TDEs by decreasing the total number of spikes 2–4 times (Figure 12), which proportionally decreases energy consumption when implemented on neuromorphic hardware (Caccavella et al., 2023).

Although we obtained a principal result regarding the benefits of inhibitory input for the performance of the TDE, there are two issues that must be addressed to secure optimal performance of TDE-3: jitter in event timing (Czech and Orchard, 2016; Ding et al., 2023) and the way inhibition is implemented.

Jitter creates a problem for direction selectivity because the reset of the gain during motion in the orthogonal direction requires simultaneous activation of facilitator and inhibitor compartments (see Sections 2, 5.2), which is hardly achievable with real-world data due to jitter. A solution to this problem is to low-pass filter inputs to the facilitator and inhibitor compartments. We did this crudely, by binning events into timesteps [which is still compatible with digital neuromorphic processors such as Loihi (Davies et al., 2018)], but the TDE could benefit from the incorporation of an adaptive low-pass filter with trainable parameters. It is crucial for the filter to be adaptive because there is a trade-off between velocity resolution, which requires short time constants, and combating noise, which requires long time constants. Adaptability is crucial since the optimal time constant depends on the particular characteristics of the visual scene such as velocity distribution, illumination, and spatial frequency.

In our approach, inhibition was implemented as resetting the gain to zero. Essentially, the same was also done by ROS et al. (1999). However, this is not the only way inhibition can be implemented. For example, in the insect T4/T5 elementary motion detectors that served as the inspiration for TDE-3, activation of the inhibitory input opens chloride channels that (i) hyperpolarize the neuron to terminate response to the stimulus and (ii) decrease membrane resistance (neuronal gain), such that the subsequent activation of what we call here the “trigger” does not have any effect on the response (Borst et al., 2020; Zavatone-Veth et al., 2020). In some biologically motivated studies, detector output was proportional to the difference between inputs to facilitator and inhibitor (Bahroun et al., 2019; Mano et al., 2021) and inputs were low-pass filtered (Mano et al., 2021). In other neuromorphic approaches, the absence of inhibition was required for a detector to produce an output Kramer (1996), but inhibition also had a terminating/subtractive effect on the detector output (Kramer, 1996; Higgins et al., 1999; Deutschmann et al., 1997). Similarly, inhibition terminated output in a two-point detector proposed in Giulioni et al. (2016).

In our opinion, the way inhibition should be implemented depends on the nature of signal coding in a given detector. For example, in insect T4/T5 cells output is encoded with graded (analog) signals that have their own low-pass filter dynamics (Borst et al., 2010; Zavatone-Veth et al., 2020). With such graded outputs, it is beneficial for inhibition to directly terminate output, because otherwise downstream neurons would be left with the impression that some slower moving edges follow a fast one. In detectors where stimulus velocity is encoded with output duration, whether as a step response (Kramer, 1996) or a spike train (Giulioni et al., 2016), inhibition directly terminating the output is appropriate. Note, however, that encoding velocity with spiking duration requires sustained high-rate spiking that is detrimental for circuitry energy efficiency and runs counter to sparsity–one of the tenets of computation in SNNs.

In spiking detectors like TDE-3 or the one presented by ROS et al. (1999), where velocity is encoded with output spike rate/interspike interval, shutting down spiking would decrease the detector's velocity coding range. Hence, to maintain coding range while avoiding responses to stimuli moving in the non-PD, it is beneficial to decouple the detector's output from inhibition.

Also note that the nature of inhibition determines between which points of a three-point detector motion is measured. When inhibition directly affects neuronal output, the motion signal is measured from trigger to inhibitor. When inhibition is decoupled from the output, we effectively measure the motion signal from facilitator to trigger. In cases where motion in PD and ND is pooled with different signs (Bahroun et al., 2019; Mano et al., 2021; Borst et al., 2010; Zavatone-Veth et al., 2020), the excitatory signal is measured between facilitator and trigger, while the inhibitory signal is measured between inhibitor and trigger.

### 4.2 Velocity inference

For the velocity inference, we developed a procedure for supervised training of the TDEs to linearly encode input velocities using spike count- and ISI-based inference (Figures 3, 4). For the ISI-based training, we developed a novel approach. While most other methods (Bohte et al., 2002; Mostafa, 2018; Comsa et al., 2020) model the relationship between input magnitude and a differentiable function then use BPTT, we measure ISI from the amplitude of the low-pass filtered spike train to use BPTT with a surrogate gradient (see Section 5.2.2 for the details). The theoretical advantage of our method is that it does not require estimation of the relationship between spike timing and input magnitude, allowing it to work with more conventional activation functions.

We investigated the performance of both inference methods in conditions with wide (Figure 5) and narrow (Figure 6) dynamic ranges of input velocities and tested their robustness toward variation in stimulus spatial frequency (Figure 9) and background activity noise (Figure 7). We found that although in a noise-free environment ISI-based inference performs slightly better than spike count in terms of the correlation coefficient between true and estimated velocity (Figures 5, 6, 9), when noise is present, spike count turns out to be more reliable, especially when both training and test inference are count-based (Figures 7, 8).

Overall, we found that the TDE-based networks are robust across a wide range of noise conditions, showing FTA of >95% and correlation with stimulus velocity of ≈90% at all noise levels except the two highest (5 Hz/px and 10 Hz/px, Figure 8). However, we suspect that in those conditions it may be possible to exploit TDEs more efficiently. To this end, we suggest more aggressive filtering at the STCF stage, as was done for the real-world data (Figures 10, 11).

Examining training progression, we observe that while the loss curves for training with spike count inference tend to be relatively smooth, the loss curves for training with ISI exhibit noticeable jumps, with changes in the loss value appearing more pulsatile than gradual. This behavior can be attributed to the initialization, which results in very high spiking rates (see Section 5.2 for the details). Under these conditions, it is easier to regulate spike count than the ISI (inter-spike interval) between the first two spikes. For spike count, improving the loss function requires only a slight adjustment in the spike count for one of the stimulus velocities. In contrast, ISI regulation is more challenging because the ISI tends to decrease over time (being shortest immediately after trigger activation). Consequently, training with ISI requires more epochs to produce meaningful differences in velocity estimation, which also manifest in a more abrupt fashion.

To decode stimulus velocity from TDE activity in the presence of multiple edges, one needs to perform motion segmentation to relate counted spikes/measured ISIs to a particular moving edge. How does one know when to count spikes or measure ISI? Here, we proposed counting spikes starting from each activation of the TDE's trigger, as it indicates the passage of a new edge. This worked for a shallow network where we could read out trigger activations, but suppose there were more layers after the TDE. How could downstream circuitry perform motion segmentation? One obvious solution would be to establish so-called skip connections between the input to the TDE's trigger and downstream circuitry. Another solution could exploit the fact that immediately after activation of the trigger, the TDE's signal is maximal; i.e., if one counts spikes within a certain sliding time window, the local (in time) maximal count would be close to the moment of edge appearance at the TDE's trigger. Similarly, the shortest distance between two spikes would occur for the first two spikes after trigger activation. Hence, downstream circuitry could potentially perform motion segmentation by performing temporal max pooling.

For velocity coding, the fundamental limitation of the TDEs is the latency vs. dynamic range dilemma (see Section 2). Since spike amplitude is usually fixed, high dynamic range requires a lot of spikes (or timepoints between spikes), which necessarily leads to a long latency in velocity inference. The best way to solve this problem is to implement TDE on a neuromorphic chip that allows spikes of different amplitudes, such as Loihi 2 (Orchard et al., 2021; Chiavazza et al., 2023), Tianjic (Pei et al., 2019), or Neuron-Flow (Moreira et al., 2020). As a result, one does not need to count spikes or intervals between spikes, because velocity magnitude is immediately available from the first spike. This would also allow simplifying the neuronal model, since the “current” and “voltage” compartments of the TDE are needed only to convert gain decay into spikes. If one can directly read out the decay value at the moment of trigger, there will be no need for such conversion. For more conventional neuromorphic hardware that produces binary spikes, one can solve the latency vs. dynamic range dilemma by employing the so-called “eccentric downsampling” using several detectors tuned to different velocity bandwidths such that each detector emits only a few spikes [Figure 15, D'Angelo et al. (2020)].

### 4.3 Performance with real-world data

We evaluated the performance of TDE-2 and TDE-3 on real-world event-based data at both the local (Figures 10, 11, Tables 3, 4) and global (ego-) motion levels (Figure 13, Tables 5, 6). Our results showed that, for both types of tasks, TDE-2 and TDE-3 achieved similar precision (Tables 3–5). However, TDE-3-based networks emit approximately 2.7 times fewer spikes than TDE-2 networks and are therefore considerably more energy-efficient. For local motion, our results show that across an 80-fold change in velocity magnitude (AEE, Table 3) and a 360° change in direction (AAE, Table 3), the performance of shallow TDE-based networks is similar to model-based local motion detectors (Rueckauer and Delbruck, 2016), but with the advantage of being naturally compatible with SNNs. Thus, they can be implemented in low-power neuromorphic hardware to exploit the spatio-temporal sparsity of event-based datastreams. For ego-motion, TDE-based inference of yaw rotations from MVSEC driving sequences performs worse than IMU but on par with neural networks containing 1.5 × 10^5^ trainable parameters (Table 5).

In the experiments with real-world data, we employed TDEs whose time constants and synaptic weights were trained on artificial data. Although this approach is theoretically prone to overfitting and a simulation-reality gap, we do not consider it a problem here. The reason is that training is used only to tune TDE parameters so that they linearly encode a certain range of velocities. In this setting, the only problem that can arise during transfer from simulation to real-world is that the real-world velocity range differs from that envisioned during training. However, since we train TDEs to encode motion in abstract terms of the fraction of the distance between facilitator and trigger covered in one algorithmic timestep (see Section 5.2.3), one can scale the dynamic range of encoded velocities by simply changing the timestep duration. Alternatively, one can employ a collection of TDEs with different distances between inputs to cover a high dynamic range of input velocities.

Despite the high correlation between TDE outputs and local optical flow (Figures 10, 11, Table 3), the absolute error in optical flow estimation remains significant—about 40%. The main sources of this error are noise, texture-induced biases, and the poor velocity resolution of individual TDEs. One way to reduce the error would be to better combine detector outputs. At present, we simply subtract outputs of oppositely tuned detectors and employ spike count/ISI to infer velocity. More sophisticated methods of spike processing–such as combining spikes across spatial locations, integrating orthogonally oriented TDEs, or jointly using spike count and ISI–might yield better estimates of optical flow. We think the most promising approach is to use trainable SNNs that take TDE outputs as inputs, ideally with self-supervised training methods that employ information bottleneck theory to ensure robust and energy-efficient processing (Yang et al., 2025).

In principle, ego-motion can be estimated by globally integrating and combining signals from differently tuned TDEs. However, the estimate would be biased by the amount of texture within a visual scene, since a few fast-moving objects may produce fewer spikes than many slowly moving objects. A solution to this problem could be some form of normalization that accounts for scene spatial frequency. Yet, our small-scale tests suggest that naive solutions, such as averaging all non-zero local velocity estimates, do not work well (data not shown). It may therefore be beneficial to train an SNN to correct for texture bias in TDE outputs.

Overall, we believe there is great potential in using TDE-3 outputs as inputs to SNNs that estimate optical flow from event-based data. TDE-3 extracts simple, abstract features such as moving edges, which are ubiquitous in visual scenes. Despite noise and biases, motion information is present in TDE outputs, and the goal of downstream processing is to extract this information by properly accounting for those imperfections. Neural networks are well suited to this task, as they can be trained to compensate for noise and bias. Indeed, combining a model-based prior for coarse estimation with a neural network for refinement is a popular approach across fields. In physics, for example, it helps accelerate convergence while reducing model complexity (Zia et al., 2025). Similarly, research on drone acrobatics has shown that autopilots trained on abstract representations of visual scenes bridge the simulation-to-reality gap more effectively than those trained on raw images (Kaufmann et al., 2020).

To conclude, we presented TDE-3 as a potent computational prior for motion detection from event-based data in SNNs. It can be trained and enables fast, efficient, precise, and robust coding of optical flow. We think that for low-level visual processing, TDE-3 offers greater robustness than Convolutional Neural Networks (CNNs). Unlike CNNs, which rely on architectures shaped by training data and are therefore prone to overfitting, TDE-3 employs a fixed, analytic architecture, reducing the risk of overfitting and enhancing stability. More broadly, our study highlights the importance of understanding biologically relevant features (e.g., the relation between structure and function) in the design of neuromorphic solutions for real-time, low-power motion detection.

## 5 Methods

### 5.1 Datasets

#### 5.1.1 Synthetic data

The synthetic dataset consists of vertically oriented bars of three light intensity values: white, gray, and black. The grayscale values were converted into events using a simplistic custom-made simulator of an event-based camera, which retained its key property: encoding changes in grayscale value with spiking signals that report increases (on events) or decreases (off events) in light intensity. Specifically, we modeled voltage in the event-based detector as being proportional to the logarithm of light intensity and reported contrast each time the temporal derivative of this signal (*V*_*t*_−*V*_*t*−1_) was higher/lower than the threshold value of ±0.15.

To compare the robustness in direction-selectivity of TDE-3 and TDE-2, we simulated their responses to moving textured stimuli consisting of bars oriented orthogonally toward the direction of motion. The bars had three intensity levels: white, gray, and black. The size of the texture along the motion axis was 80 pixels and 3 pixels along the orthogonal direction (Figure 2A). We employed five velocities: 0.1 px/timestep, 0.2 px/timestep, 0.33 px/timestep, 0.5 px/timestep and 1 px/timestep. The motion direction (left-right, right-left, top-bottom, bottom-top) and velocity were randomly chosen for each stimulus example (2,000 per testing round). To vary the “amount” of texture in stimuli we randomly varied the fraction of the gray bars from 0% to 80%.

To train the TDE-3 to linearly map velocities of moving edges into spike count-based or ISI-based output, we employed isolated edges on a homogeneous background. To avoid over-fitting and the necessity to separate stimuli into training and test datasets, for each training epoch we de-novo randomly generated 100 examples of moving edges with various velocities. We employed two sets of velocities. One was relatively wide (5 velocities spanning a 10-fold range, see above). The other was relatively narrow with 15 velocities within a range from 0.025 to 0.04 px/timestep. This stimulus set was employed to show that the TDE can encode velocities with high precision.

To study the effect of variation in scene spatial frequency on the coding precision of ISI- and spike count-based inference, we trained the TDEs to linearly encode velocities of two edges moving at the same velocities at different distances from each other. We used 5 velocities from 0.1 to 1 px/timestep and randomly varied the distance between edges within a range of 3,4,5,7 and 10 pixels.

To study the effect of noise on training and inference with spike count and ISI-based methods, we simulated one of the most prominent types of noise in event-based vision - background activity noise, as a Poisson process following the approach by Guo and Delbruck (2023). The noise was injected after the conversion of grayscale values to events as some stochastic events. We used six mean rates of noise: 0.2 Hz/px, 0.5 Hz/px, 1 Hz/px, 2 Hz/px, 5 Hz/px, and 10 Hz/px. This effectively covered the dynamic range in event-based vision from high to low SNR conditions (Guo and Delbruck, 2023). For this set of experiments, we employed the same set of 5 velocities as above and varied the distance between edges within a range of 3 to 10 pixels as above. The timestep duration was set to 10ms.

#### 5.1.2 Real-world data

We used three real-world datasets: disk and boxes recorded (Rueckauer and Delbruck, 2016) (Figures 10, 11), MVSEC driving sequences (Zhu et al., 2018b) [Figure 13, and EVIMO-2 Burner et al. (2022), Figure 12].

In Rueckauer and Delbruck (2016), data was collected with an event-based camera DAVIS 240C with a resolution of 240 × 180 pixels and contained various sequences of the full-field motion with a duration between 1 second and 4 seconds. We were specifically interested in two sequences: one with the apparent motion of the various boxes to the right (Figure 10) and the one with the disk rotating clockwise (Figure 11). The former sequence contained multiple moving edges closely following each other with very similar velocities and thus directly targeted the key weakness of the TDE-2 of loss in direction-selectivity in a textured environment (Figures 1, 2). The second sequence contained a large dynamic range of velocities (proportional to the distance from the center of rotation) and was thus ideally suited to test the optic flow coding properties of the TDEs.

The dataset contained ground truth optical flow that could be inferred from the IMU measurements. Indeed, motion in the sequences was confined to the motion of the camera: for the boxes to yaw and for the disk to roll rotations (z-axis). Hence, in this case, ground truth optical flow can be obtained as a displacement vector of individual events from the IMU measurements with Equation 1 following Rueckauer and Delbruck (2016):


(1) $$\begin{array}{l} {\text{e}}^{\prime }=\text{R}(\text{e}-{\text{e}}_{0}+\text{T})+{\text{e}}_{0} \end{array}$$

where **e** is the initial location of an event (*e*_*x*_, *e*_*y*_), **e′** - end location of the event, **e**_0_ is the location of IMU center, **T** describes motion due to yaw and pitch rotations, and **R** describes motion due to roll rotation (z-axis):


(2) $$\begin{array}{l} \text{T}=k\left[\begin{array}{c} y\\ x \end{array}\right];\;\text{R}=\left[\begin{array}{cc} \cos z & -\sin z\\ \sin z & \cos z \end{array}\right] \end{array}$$

where x,y, and z are the rotation rates of IMU around corresponding axes (pitch, yaw, and roll rotations) and k is a scaling factor that converts degrees to pixels for the yaw and pitch rotations equal to 4.25 px/° (Rueckauer and Delbruck, 2016).

The driving sequences of a car driving through West Philadelphia from the MVSEC dataset were recorded with a DAVIS 346B with resolution of 346 × 360 (Zhu et al., 2018b). We specifically were interested in four sequences: outdoor_day_1, outdoor_day_2, outdoor_night_2, outdoor_night_3. The duration of the outdoor_day_2 sequence is 10 minutes long; other sequences are about 5 minutes long. The driving sequences were accompanied by ground-truth angular velocity estimated through fusion of IMU, GPS, stereo-camera and LiDAR (Zhu et al., 2018b) and were used to test ego-motion estimation with TDEs.

The EVIMO-2 dataset was recorded with a Samsung DVS G3 with resolution of 640 × 480 and contains various scenes of indoor motion (Burner et al., 2022). We used sequences from the SFM and IMO subsets to compare energy efficiency of TDE-2 and TDE-3 (Figure 12).

### 5.2 Inference and training

#### 5.2.1 Training and inference with the synthetic data

The SNNs were simulated using the PyTorch framework, inspired by previous implementations (Neftci et al., 2019; Hagenaars et al., 2021). Depending on the application, the networks included several types of elements such as TDEs, STCFs, and low-pass filters of the spike trains (i.e., spike traces). Each element was defined by its synaptic weights and time constants.

Algorithm 1 shows the computations performed when TDE-2 and TDE-3 process input signals. Apart from the inhibitor that resets TDE-3's gain upon activation, there is no difference in signal processing. As one can see, the neuronal dynamics of TDEs resemble those of a CuBa LIF neuron. We opted for this neuronal model because of its simplicity and because TDE-3 was developed from TDE-2, which already employed the CuBa LIF neuron (Milde et al., 2018). The CuBa LIF neuron is the simplest configuration that allows conversion of the gain activation value into a spike train. In principle, other, more complicated neuronal models are possible (Gerstner et al., 2014). However, hardware implementations with graded spikes (Orchard et al., 2021; Pei et al., 2019; Moreira et al., 2020) would allow for an even simpler model than the CuBa LIF neuron, since the gain activation value could then be read out directly.

**Algorithm 1:** TDE-2 and TDE-3 neural dynamics.

Parameters:
1: *g*= gain, *i*= current, *v*= voltage, *s*= spike
2: τ= time constant, *w*_*g*_= synaptic weight, θ= threshold
3: $\alpha_{g}=\frac{dt}{\tau_{g}+dt}=\sigma \left(p_{g}\right)$
4: $\alpha_{i}=\frac{dt}{\tau_{i}+dt}=\sigma \left(p_{i}\right)$
5: $\alpha_{v}=\frac{dt}{\tau_{v}+dt}=\sigma \left(p_{v}\right)$
TDE-2
1: for *t*∈[1, *T*] **do**
2: *i*[*t*]←α_*i*_·*i*[*t*−1]+*g*[*t*−1]·*Tr*[*t*]
3: *g*[*t*]←α_*g*_·*g*[*t*−1]+*w*_*g*_·*Fac*[*t*]
4: *v*[*t*]←α_*v*_·*v*[*t*−1]+*i*[*t*]
5: *s*[*t*]←*spike*_*fn*(*v*[*t*]−θ)
6: *v*[*t*]←(1−*s*[*t*])·*v*[*t*]
7: end **for**
TDE-3
1: for *t*∈[1, *T*] **do**
2: *i*[*t*]←α_*i*_·*i*[*t*−1]+*g*[*t*−1]·*Tr*[*t*]
3: *g*[*t*]←(α_*g*_·*g*[*t*−1]+*w*_*g*_·*Fac*[*t*])·(1−*Inh*[*t*])
4: *v*[*t*]←α_*v*_·*v*[*t*−1]+*i*[*t*]
5: *s*[*t*]←*spike*_*fn*(*v*[*t*]−θ)
6: *v*[*t*]←(1−*s*[*t*])·*v*[*t*]
7: end **for**

The trainable parameters of the TDE included the gain, current, and voltage time constants, as well as the synaptic weight of the gain compartment. To facilitate learning of the neuronal time constants, we used the approach proposed by Fang et al. (2021), which reformulates neuronal decays as the product of the gain, current, and voltage with the sigmoid of a trainable parameter (*p*_*g*_, *p*_*i*_, *p*_*v*_).

To mitigate the effect of background activity noise in experiments with real-world data and injected noise, we employed STCF filtering as described by Guo and Delbruck (2023) (Algorithm 2). In this simple yet effective algorithm, an event is allowed to pass the filtering stage only if at least one event occurs in the *n* nearest neighboring pixels (a trainable parameter) within a specified time window. Essentially, the filter extracts correlations (hence the name) between a pixel and its neighborhood while rejecting uncorrelated noisy events. As a result, STCF improves the signal-to-noise ratio while preserving signal sparsity. The filter can be effectively modeled as an LIF neuron with a very short leak constant, whose firing threshold is parameterized by the number of neighboring pixels *n*.

**Algorithm 2:** STCF implementation.

Parameters/state:
1: *v*[*x, y, t*]= filter voltage at pixel (*x, y*) and time *t*
2: *s*[*x, y, t*]= filter output spike at pixel (*x, y*) and time *t*
3: *e*(*x, y, t, p*)= input events with polarity *p*
4: *n*= spike threshold
5: for *t*∈[1, *T*] **do**
6: for each pixel (*x, y*) **do**
7: $v\left[x,y,t\right]\leftarrow \underset{p}{\sum }\overset{1}{\underset{i=-1}{\sum }}\overset{1}{\underset{j=-1}{\sum }}e\left(x+i,y+j,t,p\right)$
8: *s*[*x, y, t*]←spike_fn(*v*[*x, y, t*]−*n*)
9: end **for**
10: end **for**

**Figure 16:**
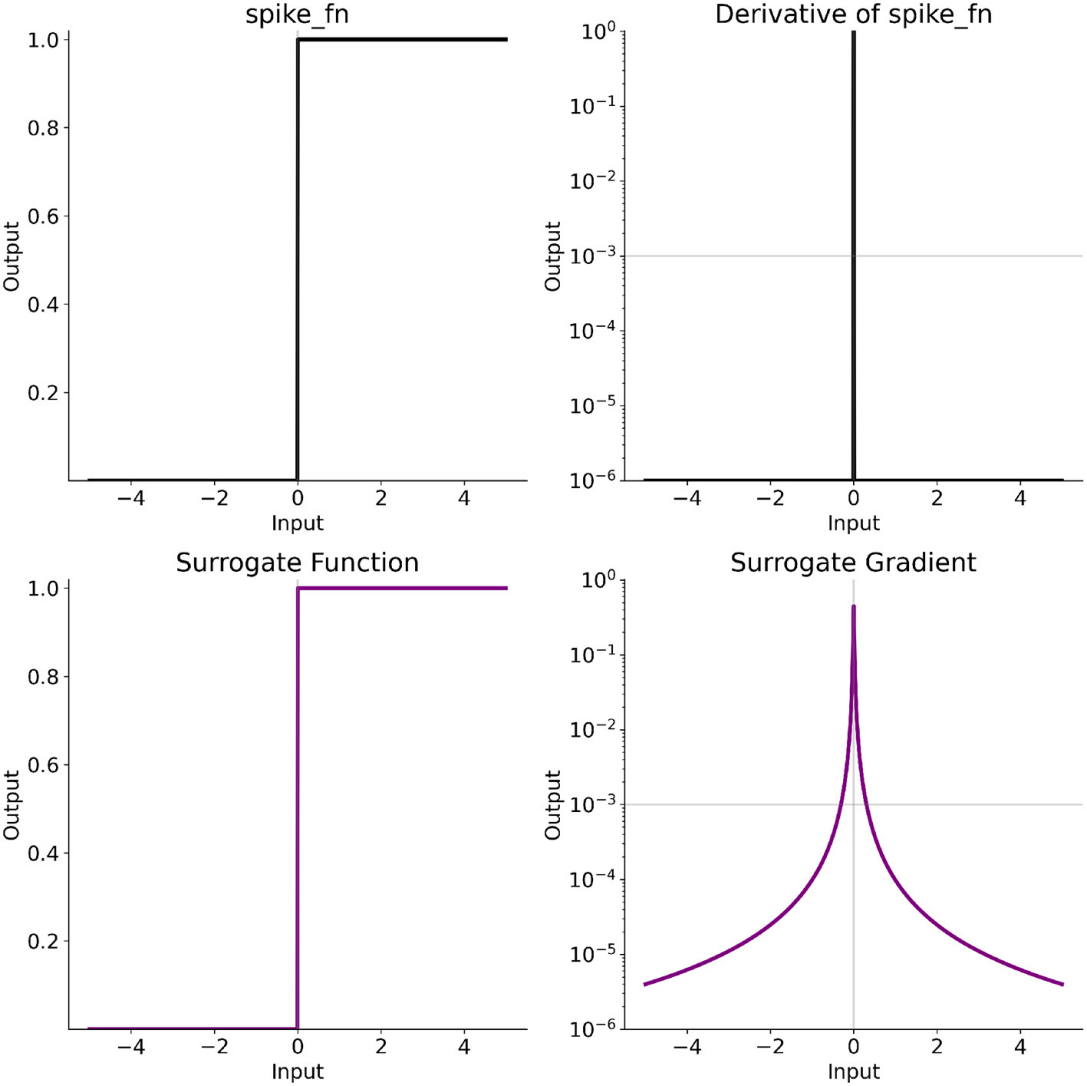
Surrogate gradient.Top, left—spiking (Heaviside) function. Top, right—derivative of the spiking function (Dirac function). Bottom, left—surrogate function. Bottom, right—gradient of the surrogate function that was used during backward pass. Note logarithmic scale for the derivative plots.

To train SNN with BPTT, we addressed the non-differentiability of the spiking activation function by using surrogate gradients (Neftci et al., 2019; Zenke and Vogels, 2021) (Figure 16). This method enables backpropagation in SNNs by replacing the spiking activation function with a differentiable function of similar shape and steep slope during the backward pass (Neftci et al., 2019; Zenke, 2019; Zenke and Vogels, 2021). We used the surrogate gradient proposed in Zenke (2019) (Figure 16). The right column of Figure 16 compares the derivative of the spiking function (i.e., the Heaviside function) with the surrogate gradient function on a logarithmic scale. As shown, while the derivative of the spiking function is zero everywhere except at 0, the surrogate gradient has a small non-zero derivative, which allows the error gradient to pass and thus enables training of the SNNs.

Depending on the coding scheme (spike count or ISI), we used two slightly different training pipelines to extract motion direction and velocity (Figures 3, 4). The two first steps are common to both pipelines. The input layer simply converts events to spikes. Then, these spikes are fed into the layer of TDE-3s, which effectively perform 1D convolution with kernel size=3, stride=1, and weights being facilitator, trigger, and inhibitor. This layer consists of two channels, one for each motion direction. At the next processing stage, spike count-based and ISI-based pipelines deviate. For the spike count-based velocity estimator we can straightaway find the total number of spikes emitted by each of the detectors, convert it to estimated velocity (see Algorithm 3), calculate the loss, and perform a backward pass. For the ISI-based velocity estimation, the training of the network is not so straightforward and is described in Section 5.2.2.

**Algorithm 3:** Velocity inference with spike count.

Parameters/state:
1: *win*= temporal window over which spikes are integrated
2: β= factor to scale the spike sum
3: *i*_tde_[*t*]= current in the corresponding TDE
4: *i*_inc_= indicator of whether TDE's current increased
5: *s*_tde_[*t*]= spikes from a TDE
6: *V*[*t*]= velocity estimate
7: for *t*∈[1, *T*] **do**
8: *i*_inc_[*t*]←spike_fn(*i*_tde_[*t*]−*i*_tde_[*t*−1])
9: $V\left[t\right]\leftarrow \beta \cdot \overset{0}{\underset{k=-win}{\sum }}s_{\text{tde}}\left[t+k\right]\cdot i_{\text{inc}}\left[t-win\right]$
10: end **for**

To promote linear mapping of input velocity into velocity estimated from TDE-3's output activity, we used the mean absolute error (L1 loss) between the normalized TDE-3 estimates and the ground truth edge velocities. Normalization ensures linear mapping because it removes scale such that the loss depends only on the nonlinearities of the mapping. Normalization was done with respect to the maximum estimate/velocity in the batch (Equation 3). Additionally, to ensure the sparsity of the learned solution, we added a regularization loss which is proportional to the mean squared spike count. Spikes were summed along temporal dimension and average across examples within the batch (Equation 3). At the same time, to avoid the vanishing of the gradient due to low neuronal activity (Rossbroich et al., 2022), the network was initialized to have a high firing activity by making the synaptic weights large and the time constants long.


(3) $$\begin{array}{l} L_{\text{train}}=\underset{\text{L1 velocity loss}}{\underset{︸}{\frac{1}{N}\overset{N}{\underset{b=1}{\sum }}\left|{\hat{v}}_{\text{est},b}-{\hat{v}}_{\text{true},b}\right|}}+\\ \;\underset{\text{regularization loss}}{\underset{︸}{0.05{\left(10^{-2}\cdot \frac{1}{N}\overset{N}{\underset{b=1}{\sum }}{\left(\overset{T}{\underset{t=1}{\sum }}s_{\text{tde}}\left(b,t\right)\right)}^{2}\right)}^{\frac{1}{2}}}} \end{array}$$

For the velocity set with a wide (10-fold) dynamic range of velocities, the detector is trained to linearly map this dynamic range into limited spiking output. Hence it makes sense to estimate velocity as a spike count scaled by minimal discernible velocity (0.1 px/timestep), such that minimal velocity is encoded with one spike. For the narrow dynamic range (1.5-fold), the detector is trained to encode a limited dynamic range with very high precision. Hence, it makes sense to use a bias factor: to infer velocity, we multiply the spike count by the velocity resolution (0.001 px/timestep), and then, if the spike count is larger than 1, we add to it a bias factor of 0.024 px/timestep. This bias factor is necessary because it allows encoding a velocity of 0.025 px/timestep with only 1 spike. Without this bias factor to have a velocity resolution of 0.001 px/timestep, would have required to use of 25 spikes for the velocity of 0.025 px/timestep.

To assess TDE-3 performance in the presence of multiple moving edges, one needs to perform motion segmentation: to relate counted spikes to particular edges. To do so we (1) limit the temporal window within which spikes are counted to 10 timesteps and (2) count spikes starting from the increase in TDE current. An increase in current is always a consequence of activation of the trigger. This means that the motion of a new edge causes it and thus solves the problem of motion segmentation. When we trained the detector to encode velocity amidst variation in stimulus spatial frequency, we calculated the error for the velocity estimate of each of the edges.

In the experiments with noise (Figures 7, 8), not every increase in current is caused by a moving edge, and one of the goals of the training is to suppress the TDE activations caused by noise. To account for this factor in the calculation of the loss, we calculated the loss between all of the velocity estimates performed by the TDE and stimulus ground truth at corresponding timepoints (effectively, this ground truth was zero everywhere except at the moment of edge appearance see Section 5.1). Also, to evaluate how the trained TDE can reject noise and preserve the true motion signal, we employed an additional metric called fraction of true activity (FTA) as the sum of estimated velocities caused by the stimulus (true motion signal) to the total sum of estimated velocities (caused by signal and noise).

#### 5.2.2 Training of the spiking neural networks to code with interspike interval

Apart from spike count, another intuitive method to encode signals in SNNs is relative spike timing (Gollisch and Meister, 2008; Rieke et al., 1999). The main benefit of such signal coding is that it allows for much shorter latency (Rieke et al., 1999; Gollisch and Meister, 2008) while also enhancing coding capacity on a network level (Paugam-Moisy and Bohte, 2012). Training SNNs to have specific spike timing has a long history, with the first classical paper published as early as 2002 (Bohte et al., 2002).

In the present paper, we developed a novel method to train SNNs to encode a signal with ISI using supervised learning with BPTT (Figure 4). While most other methods (Bohte et al., 2002; Mostafa, 2018; Comsa et al., 2020) model the relationship between input magnitude with differentiable function and then use BPTT, we measure ISI from the amplitude of low-pass filtered spike train to use BPTT with surrogate gradient. The theoretical advantage of our method is that it does not require estimation of the relationship between spike timing and input magnitude, allowing to work with more conventional activation functions.

**Algorithm 4:** Estimation of velocity from ISI.

Parameters/state:
1: τ_*f*_= filter time constant
2: γ= scaling factor
3: *x*_0_= spike trace amplitude upon arrival of the first spike
4: *i*_inc_= indicator of whether TDE's current increased
5: *x*[*t*]= spike trace at time *t*
6: *v*_est_= estimated velocity
7: for each *i*_inc_>0 **do**
8: Take filter output *x* at the timestep preceding the second spike since increase in current
9: Estimate elapsed time since first spike: $t\leftarrow \tau \cdot \ln\left(\frac{x_{0}}{x}\right)$
10: Estimate inter-spike interval: *ISI*←*t*+1
11: Estimate velocity: $v_{\text{est}}\leftarrow \frac{\gamma }{ISI}$
12: end **for**

Specifically, we were interested in the ISI between the first two spikes, as it should provide the shortest latency (Milde et al., 2018; Gutierrez-Galan et al., 2022). The key is to estimate the time between two spikes in a differentiable manner. To do so, we added to the pipeline in Figure 3 low-pass filtering of the TDE output spikes (Figure 4).The filtering yields an exponentially decaying spike trace *x*(*t*). By taking filter output at the timestep directly preceding the second spike one can estimate ISI as *ISI* = *t*(*x*)+1. Next, by taking an inverse of it and scaling it by a proper factor (γ) one can convert it to the estimate of the input velocity. Finally, to train a network with BPTT one can calculate the loss function using Equation 3.

On the practical level, there are three factors to take care of to successfully implement a training pipeline based on the ISI:

1. ISI interval is undefined when the TDE emits less than two spikes because the notion of interval requires 2 points. We assume that < 2 TDE spikes mean an extremely slow motion of an object. Therefore we set ISI to a very large value (10^3^ - 10^6^ timesteps) such that when inverted it will lead to an extremely low estimated velocity.
2. Scaling of the inverted ISI to estimate velocities. This can be done similarly to scaling of spike count: for the stimuli set with a wide dynamic range we multiplied the maximal inverted ISI (=1) with maximal stimuli velocity; for the stimuli set with a narrow dynamic range, we also used bias and multiplied inverted spike count with the difference between maximal and minimal stimuli velocity.
3. Handling of multiple moving edges. We opted to handle multiple moving edges similarly to spike count inference: the first two spikes were determined with respect to the current increase. Also, in the presence of noise, the loss function and performance evaluation were handled similarly to the spike count.

#### 5.2.3 Nuances of the application of TDEs to the real-world data

Real-world data was used only as a test dataset. We pooled together ON and OFF events as it is often encountered in biological systems (Clark and Demb, 2016; Borst et al., 2020; Sterling and Laughlin, 2015). The network was trained with synthetic moving bars to fit its dynamic range to the dynamic range of velocities in real-world data. Compared to simulation, estimation of the optical flow with TDEs in the real-world data has three additional challenges: (1) other (not only background activity) types of noise, (2) the necessity to coordinate the activity of multiple TDEs, and (3) the high dynamic range of velocities (2 decades for the rotating disk).

Apart from background activity noise, another prominent source of noise in the event-based data is jitter in event timing (Czech and Orchard, 2016). Event arrival is specified with microsecond precision, yet the time between actual change in intensity and event generation can vary in a range of milliseconds. Therefore, strictly speaking, each pixel of a moving edge generates an event at a different time (Czech and Orchard, 2016). This is especially problematic for the 3-point TDE because the prevention of its activation upon motion in an orthogonal direction is underpinned by simultaneous activation of facilitator and inhibitor, which is hardly possible given the event jitter. To avoid this problem, one needs to increase the co-activation of the facilitator and inhibitor. The most straightforward way to do so is to downsample the event stream in time. For instance, feeding events to the network using time bins, where events within a bin are considered to occur simultaneously. There is a trade-off between mitigation of event jitter and dynamic range of velocity coding. Here (Figures 10, 11), given the relatively low stimulus velocity, we chose to slice events into bins of 50 ms (i.e. 20 frames per second). The same frame rate was chosen for the driving sequences from MVSEC dataset because there ground truth was available at 20Hz (Zhu et al., 2018b) (Figure 13). For consistensy, we also employed the same frame rate with EVIMO-2 dataset (Burner et al., 2022) (Figure 12).

In contrast to synthetic data where we mostly employed either one or two opposingly tuned detectors, with the real-world data we sampled each pixel of the visual field with TDEs tuned along 4 cardinal directions: Left-to-Right (LR), Right-to-Left (RL), Top-to-Bottom (TB), and Bottom-to-Top (BT) akin to how it is done in vertebrae and insect visual systems (Clark and Demb, 2016). Although there are 4 cardinal directions, motion occurs along 2 axes: horizontal and vertical. Since a point in space cannot simultaneously move in 2 opposite directions, the next logical step in the processing pipeline is to subtract velocity estimates obtained from opposingly tuned detectors. This move is ubiquitously employed in biological systems (Borst et al., 2020; Clark and Demb, 2016; Reichardt, 1961) and was shown to optimize velocity coding by biological elementary motion detectors (Kühn and Gollisch, 2019). Here we found that such a subtraction compensates for the poor direction-selectivity of individual TDE-2 (Tables 4–6).

The sequence with a rotating disk contained a very high dynamic range of velocities (2 decades: from 1 to 80 px/s). As was discussed earlier, using a single TDE to encode such a high dynamic range with high resolution is sub-optimal in terms of latency. Yet, it is possible to do so using a set of detectors tuned to different velocities. One elegant way to achieve so is to gradually increase spacing between TDE inputs (i.e. facilitator, trigger, inhibitor) as the detector position moves away from the center (D'Angelo et al., 2020) (Figure 15). Indeed, given internal parameters, the TDEs where the facilitator and trigger are spaced by n pixels can encode n times higher velocities than the detector where the facilitator and trigger are spaced by 1 pixel.

Although such an eccentric increase in TDE receptive fields nicely fits the rotation disk (where velocity increases as one moves out of the center), this solution is rather general and universally encountered in animal visual systems (Sterling and Laughlin, 2015). The reason for this is that most of the optical flow is engendered by self-motion and most of the self-motion is directed forward. As animals tend to look toward the focus of expansion (a point in the visual field from which optical flow originates), the further an object is from the center of the visual field, the higher its retinal velocity. Given that robots also mostly move forward, eccentric increase in the receptive field is beneficial for robotics applications as well (D'Angelo et al., 2020).

The specifics of our implementation are as follows. Using synthetic data, we first pre-trained TDE with spacing between compartments of 1 px to linearly encode 5 levels of velocity (from 0.1 px/timestep to 0.5 px/timestep). Now, it is important to recognize that the TDE encodes only the delay between activations of the facilitator and trigger compartments. Hence, the results of the training are indifferent toward the spatial configuration of the TDE, i.e. resultant velocity coding is not so much about px/timestep as about a fraction of the distance between facilitator and trigger covered within a timestep. Hence, by simply regulating the distance between the TDE inputs by a factor of *n*, one can scale the TDE range of velocity by said *n* without the need to re-train the TDE. Here, we used TDEs with distances between inputs of 1, 2, 3, 4, 6, and 8 pixels (Figure 15). Paired with a timestep duration of 50ms, it means that together the TDEs were able to cover velocities in the range of 2 to 80 px/s. For the spike count inference, the window over which spikes were counted was limited to 5 timesteps. Before feeding into the layer of TDEs signal was filtered by 3 by 3 STCF filter (Guo and Delbruck, 2023).

For the MVSEC and EVIMO-2 datasets we employed TDE with distance between inputs equal to 2. For MVSEC dataset we used LR and RL TDEs and calculated total activity by pooling spikes from LR TDEs with positive sign, and spikes from RL TDEs with negative sign.

#### 5.2.4 Performance evaluation for the real-world data

For the qualitative comparison between the optical flow inferred with the TDEs and the ground truth, we visualized the optical flow using the colormap in Figure 14. To make quantitative comparisons, we used the Angular Average Error (AAE), Average Endpoint Error (AEE), relative Average Endpoint Error (rAEE), Angular Velocity Error (AVE), Average Relative Rotation Error (Zhu et al., 2018a), and Pearson correlation coefficient (Pearson, 1895).

AAE measured the difference between the direction of motion estimated with the TDEs and the ground truth motion direction, and it was calculated with Equation 4:


(4) $$\begin{array}{l} AAE=\frac{1}{N}\overset{N}{\underset{i=1}{\sum }}\arccos\left(\frac{v_{ix}u_{ix}+v_{iy}u_{iy}}{\left|{\text{v}}_{i}||{\text{u}}_{i}\right|}\right) \end{array}$$

where **v** is the velocity vector estimated with the TDEs, **u** is the ground truth vector, and i denotes individual optical flow estimates (i.e. per pixel per timestep). Zero velocity measurements were not counted in Equation 4. AEE was calculated as the difference between optical flow vectors inferred with TDE and ground truth using Equation 5:


(5) $$\begin{array}{l} AEE=\frac{1}{N}\overset{N}{\underset{i=1}{\sum }}\sqrt{{\left(v_{ix}-u_{ix}\right)}^{2}+{\left(v_{iy}-u_{iy}\right)}^{2}} \end{array}$$

The problem of AEE is that it is hard to get from it an intuition about the quality of velocity estimate. First of all, this error accumulates over time such that it becomes hard to compare algorithms run at different simulation frequencies. Secondly, understanding whether coding error is acceptable does not depend on the absolute value of error, but on the ratio between error and true velocity. Therefore, we used rAEE to normalize AEE by the ground truth velocity to give a relative measure of performance quality using Equation 6:


(6) $$\begin{array}{l} rAEE=\frac{1}{N}\overset{N}{\underset{i=1}{\sum }}\frac{\sqrt{{\left(v_{ix}-u_{ix}\right)}^{2}+{\left(v_{iy}-u_{iy}\right)}^{2}}}{\left|{\text{u}}_{i}\right|} \end{array}$$

Angular Velocity Error was calculated in the similar way to Average Endpoint Error, such that mean AVE across the entire sequence can be calculated with equation:


(7) $$\begin{array}{l} \text{mean AVE}=\frac{1}{T}\overset{T}{\underset{t=1}{\sum }}|{\dot{\psi }}_{\text{gt}}^{\left(t\right)}-{\dot{\psi }}_{\text{est}}^{\left(t\right)}| \end{array}$$

where ${\overset{•}{\psi }}_{\text{gt}}$ and ${\overset{•}{\psi }}_{\text{est}}$ are the ground truth and estimated yaw rotation rates.

To benchmark our performance on the MVSEC dataset, we employed ARRE, which estimates the error between two rotations using the formula:


(8) $$\begin{array}{l} \text{ARRE}\left(P,G\right)=\overset{T}{\underset{t=1}{\sum }}||logm\left(P_{t}^{⊤}G_{t}\right){||}_{2} \end{array}$$

where *T* is the duration of the sequence, $P_{t}^{⊤}$ is the transpose of the predicted (estimated) orientation at time *t* (pitch, roll, and yaw), *G*_*t*_ is the ground truth orientation, and logm denotes the matrix logarithm. ARRE describes the error in ego-motion estimation according to the following reasoning.

Orientation in space can be specified using a rotation matrix *R*. One of the key properties of rotation matrices is that their inverse is equal to their transpose, i.e., *R*^−1^ = *R*^⊤^. This property allows us to estimate the difference between two rotations *R*_1_ and *R*_2_ as


(9) $$\begin{array}{l} \Delta R=R_{1}^{⊤}R_{2}, \end{array}$$

where Δ*R* is the relative rotation from *R*_1_ to *R*_2_ (Lynch and Park, 2017). Indeed, if *R*_1_ = *R*_2_, the result is the identity matrix *I*, whereas any deviation from *I* quantifies the difference between the two rotations.

Although we can calculate the error in this way, Δ*R* is still a matrix, and ideally we want a single scalar to quantify the rotational difference. Fortunately, rotations can also be parametrized in exponential form:


(10) $$\begin{array}{l} R_{2}=R_{1}\exp\left(\chi \hat{\text{ w}}\right), \end{array}$$

where $\hat{\text{w}}$ is the unit vector along the axis of rotation, and χ is the rotation angle (Lynch and Park, 2017). To extract the angle χ corresponding to the rotational difference, we take the matrix logarithm of $R_{1}^{⊤}R_{2}$. The same reasoning is applied in Equation 8 to reduce a rotation difference to a single scalar measure.

How can we obtain rotation matrices to calculate ARRE? To do so, we can integrate the ground truth angular velocity (Zhu et al., 2018b,a) as well as the angular velocity estimated by the TDEs. At first, it might seem unusual to compute rotations when both the network output and the ground truth already provide angular velocity. The reason is that we aim to compare our results with other studies, and most previous works (Ye et al., 2020; Zhu et al., 2018a; Mitrokhin et al., 2019a) employed neural networks where rotations were learned in a self-supervised fashion—by forcing the network to estimate the rotation between two images sampled at different time intervals.

Since we measure only yaw rotations and assume all other rotations are zero, the rotation axis $\hat{\text{w}}$ coincides with the yaw axis, and χ represents the yaw angle. Therefore, the ARRE reduces to a simple product of the average angular velocity error (AVE) and the sampling interval Δ*t*:


(11) $$\begin{array}{l} {\text{ARRE}}_{\text{yaw}}\approx \text{AVE}\cdot \text{Δ}t. \end{array}$$

## Data Availability

The data and code that support the findings of this study are openly available in the GitHub repository at https://github.com/yedutenko/TDE-3.

## Footnotes

^1^TDE implementation on Intel Loihi: https://github.com/intel-nrc-ecosystem/models.
