# Supplementary material for: TDE-3: an improved prior for optical flow computation in spiking neural networks
Source: Front Neurosci. 2025 Nov 3;19:1667541. doi: 10.3389/fnins.2025.1667541 (PMC12621106; doi:10.3389/fnins.2025.1667541)
Supplement: Supplementary file 1 [file Data_Sheet_1.pdf]

1 Supplementary Information

1.1 Robustness of TDE-2 and TDE-3 direction-selectivity when visual stimuli move in 2D

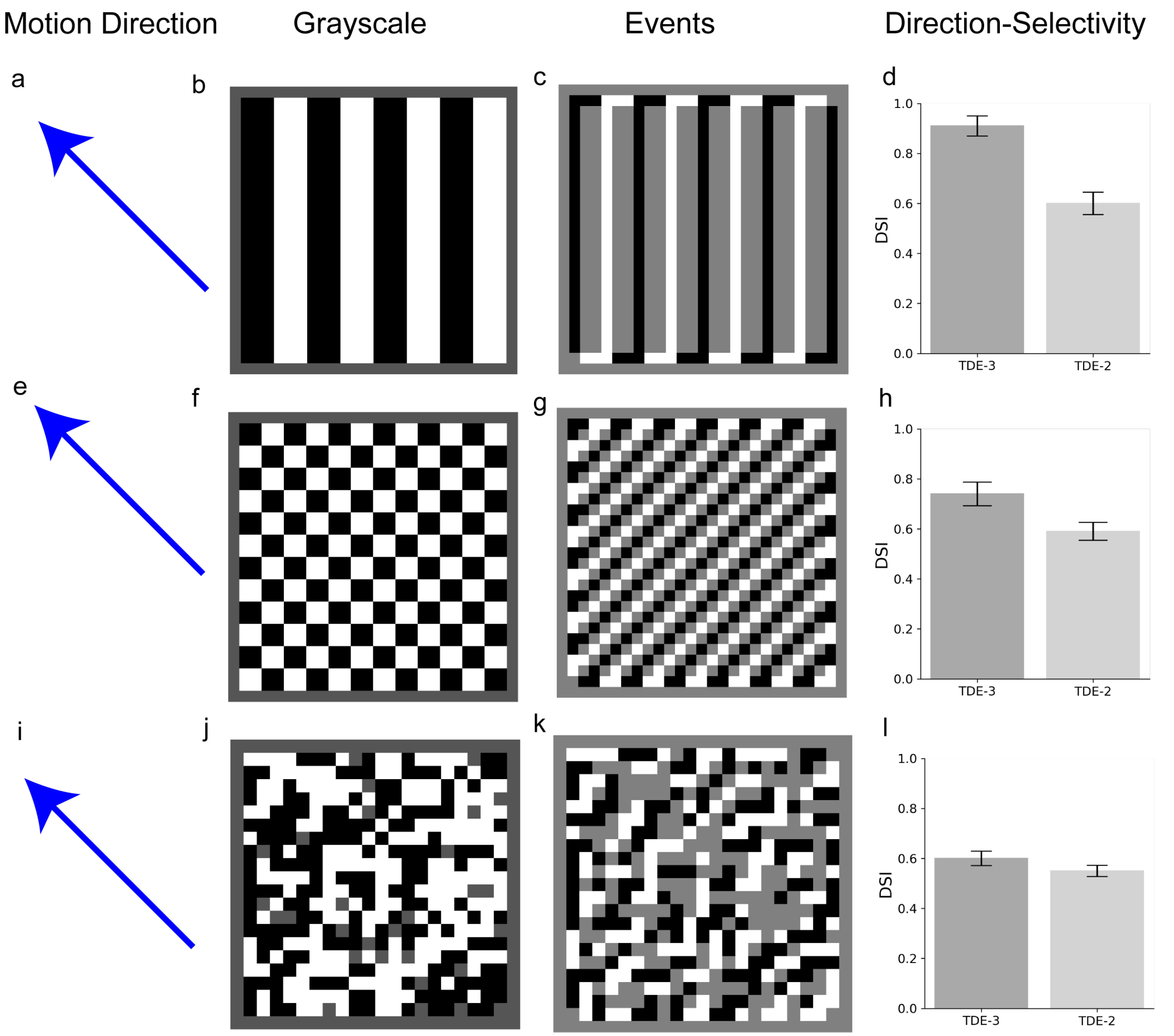

Figure S1: Direction-selectivity in presence of the stimuli moving in 2D. Columns from left to right: motion direction, grayscale images, event-based images, direction-selectivity index.

To compare robustness of TDE-2 and TDE-3 direction selectivity in case of textured stimuli moving in 2D we performed experiments with 3 types of stimuli: vertical bars (Figure S1 top row), checkerboard (Figure **??** middle row) and randomly generated texture (Figure Figure S1 bottom). The goal of these stimuli selection was to successively challenge TDE's with more and more complex patterns.

We performed 100 testing rounds with each round containing 600 examples. For each example we randomly selected velocity along horizontal and vertical axes from the range of 0.1 px/timestep, 0.2 px/timestep, 0.25 px/timestep, 0.33 px/timestep, 0.5 px/timestep and 1 px/timestep. The velocities were selected independently for each axis. For each testing round, the TDE parameters were randomly sampled to facilitate the comparison between TDE-2 and TDE-3 at the detector architecture level. All textures had a size of 24 by 24 pixels and were presented on a gray background. For the vertical bars, for each example we varied bars width in the range from 1 to 4 pixels, the size of the checkers in checkerboard stimuli was also varied in range from 1 to 4 pixels. For randomly generated textures, the percentage of gray pixels was varied in a range from 0 to 80%. Without loss of generality and for the sake of simplification of the

analysis the stimuli were moving from bottom right angle to top left angle. We focused our analysis on responses of 4 TDEs located in the center of the image and each tuned along one of the cardinal directions.

Top row of the Figure S1 illustrates experiments with vertically moving bars: b – grayscale values, c – event-based image, d – Direction Selectivity Index. Analysis of direction selectivity showed that for TDE-3 on average 91 ± 4% of the spikes were "in the correct direction" i.e. emitted by the TDEs tuned along R-L and B-T directions, while for the TDE-2 this number was 60 ± 4.5%. Thus, TDE-3 significantly (p=1.5e-67, two-sample t-test) outperformed TDE-2. However, it didn't reach 100% result. There are two reasons for this: 1) absence of TDE-3 activation in OD is underpinned by the fact that stimulus moving in OD covers all three inputs to the TDE simultaneously, which is not always the case due to motion in 2 directions and distribution of bar width; 2) absence of TDE-3 activation in ND is underpinned by the fact that motion in ND first activates inhibitor that removes residual gain activity, yet this condition is not always met in case of the 2D motion. On average, compared to TDE-2, TDE-3 emitted 3x less spikes.

Vertical bars are very simple stimuli, which also generate optical flow in 2 directions only on the edges and corners. Therefore, next we made the task more complicated by stimulating TDEs with checkerboard stimuli (middle row in Figure S1: f–grayscale values, g–event-based image, h–Direction Selectivity Index). Analysis of the direction selectivity showed that for the TDE-3 on average $74 \pm 4.7\%$ of the spikes were in the correct direction, while for the TDE-2 this number was $59 \pm 3.6\%$. Thus, TDE-3 again significantly ($p = 2.6 \times 10^{-52}$, two-sample t-test) outperformed TDE-2. To put these percentages in perspective, if there will be no actual direction-selectivity in TDE responses, then 50% of the spikes would come from detectors tuned to R–L and B–T motion. Thus, with respect to this non-selective baseline, direction-selectivity of TDE-3 is $24/9 \approx 2.67$ higher than the direction-selectivity of TDE-2. We also found that compared to TDE-2, TDE-3 on average emitted $2.3\times$ fewer spikes.

Next, we made optical flow pattern even more complicated by stimulating TDEs with moving randomly generated textures (Figure S1, j – grayscale values, k – event-based image, l – direction-selectivity index). Here, although TDE-3 was still better than TDE-2 (60 ± 2.9% vs 55 ± 2.2%, p=9.4e-34, 2-sample t-test) both detectors showed rather mediocre performance. The reason for this was that shown stimuli were too complex to correctly distinguish their motion direction from the field of view of a single TDE. Supplementary video 1 contains recording visual stimuli seen from 3 by 3 pixels window (similar to field of view of 4 TDEs each tuned to one cardinal direction) and shows that even for a human it would be hard to determine correct direction of motion. We also found that compared to TDE-2, TDE-3 emitted 40% less spikes.

To summarize, simulations of TDE responses to textures of varying complexity moving in 2D show that although TDE-3 gradually loses its direction selectivity as texture complexity increases, it always outperforms TDE-2.

1.2 Ego-motion estimation with 200 TDEs

We followed the approach of Greatorex et al. [1] and simulated the responses of TDE-2 and TDE-3 to the Outdoor_day_1 driving sequence from the MVSEC dataset [2], using a timestep of 0.1 ms to capture the continuous-time dynamics of TDE responses implemented on neuromorphic hardware. The network parameters were identical to those in [1] and are listed in Table S1. We did not use an STCF filter in this experiment, as its use was not mentioned in [1].

Table S1: Simulation parameters.

Parameter	Value
Timestep duration (dt)	0.1 ms
Synaptic weight	0.02
Time constant, gain	20 ms
Time constant, current	20 ms
Time constant, membrane	20 ms
Time constant, A	750 ms

To estimate yaw rotation rate, we selected 20 by 20 boxes centered along Y axis and offset along the X axis (blue boxes, Figure S2 top). Then we randomly positioned 100 TDEs (50 L-R tuned, 50 R-L tuned) in each of the boxes and estimated spikes by integrating outputs of these 200 detectors (L-R with a positive sign, R-L with a negative sign) following equation S1. The integrated spiking activity was then normalized to the maximal absolute value within a sequence to obtain estimate of the angular velocity.

$$\tau_A \frac{dA}{dt} = -A + \sum_i \delta(t - t_i^{LR}) - \sum_i \delta(t - t_i^{RL}), \tag{S1}$$

where the delta function $\delta(t)$ is defined as

$$\delta(t) = \begin{cases} 1, & t = 0, \\ 0, & t \neq 0. \end{cases}$$

Middle row in Figure S2 compares angular velocity estimated with TDE-2 (cyan) and TDE-3 (deeppink) with the ground truth (black) and IMU data (grey trace). Visually, except for the second turn to the left at the beginning of the sequence (keep in mind that optical flow goes in the direction opposite to the direction of movement), both TDE-2 and TDE-3 reasonably follow the ground truth. Quantitatively, the ARRE was $0.0046\,\mathrm{rad}$ for TDE-3 and $0.0049\,\mathrm{rad}$ for TDE-2, three times larger than the ARRE of the IMU data ($0.0014\,\mathrm{rad}$) and approximately 30 times larger than $0.00014\,\mathrm{rad}$ reported by Greatorex et al. [1]. Interestingly, if we integrate the angular velocity to obtain a plot of vehicle orientation over time (Figure S2, bottom), we see that the integrated error in our experiments has roughly the same magnitude ($\approx 4\,\mathrm{rad}$) as the integrated error in [1] (see their Figure 6), although with opposite sign: while in [1] the integrated orientation is overestimated, in our case it is underestimated.

How can we explain the differences in ARRE and integrated error between the present study and the work by Greatorex et al. [1]? The main differences between our study and that of Greatorex et al. [1] are the exact selection of box locations and the spatial distribution of TDEs. It is plausible that Greatorex et al. were able to place their detectors in locations with minimal texture-induced bias or noise. In small-scale tests, we observed that the exact placement of TDEs does indeed affect ARRE, although in our experiments the effect was on the order of 20% rather than 30-fold.

The difference in the sign of the integrated error can be explained by the fact that our TDE-2 and TDE-3 overestimate angular velocity during right turns (negative sign) and fail to report the second left turn. In contrast, Greatorex et al. [1] overestimated the magnitude of left turns and reported a non-existent prolonged left turn (around second 30 in their Figure 6). Note that the integrated error does not necessarily correlate with the error in angular velocity estimation, since large positive and negative errors can cancel each other during integration. For example, in some of our tests the integrated error was as low as $1.8, \mathrm{rad}$, while ARRE was $0.0055, \mathrm{rad}$.

References

[1] Hugh Greatorex, Michele Mastella, Madison Cotteret, Ole Richter, and Elisabetta Chicca. Event-based vision for egomotion estimation using precise event timing, 2025.

[2] Alex Zihao Zhu, Dinesh Thakur, Tolga Özaslan, Bernd Pfrommer, Vijay Kumar, and Kostas Daniilidis. The multivehicle stereo event camera dataset: An event camera dataset for 3d perception. *IEEE Robotics and Automation Letters*, 3(3):2032–2039, 2018.

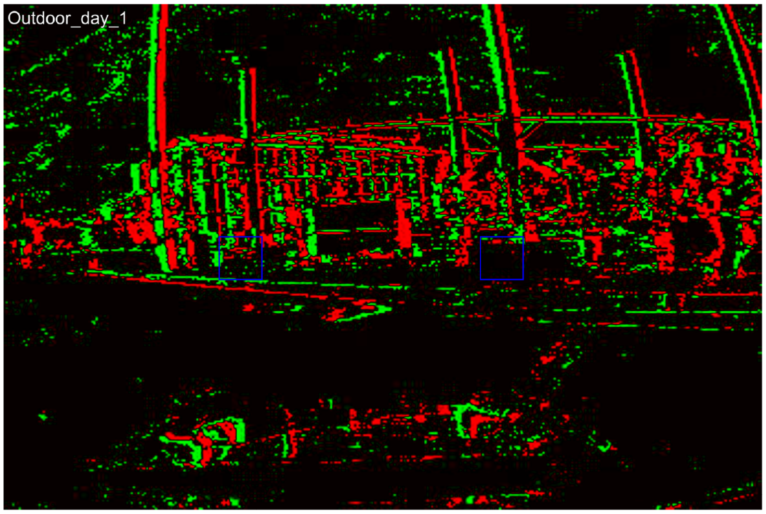

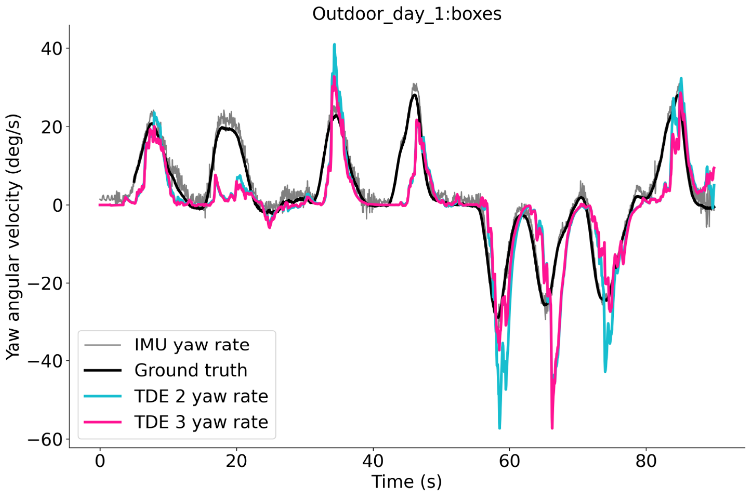

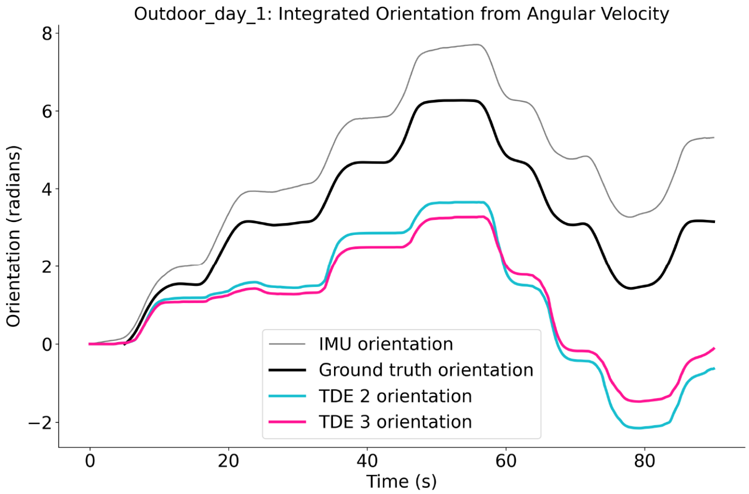

Figure S2: Inference of angular velocity with small network. Top - example of the visual scene. Middle - comparison of estimated, IMU and ground truth angular velocities. Bottom - integrated orientation error.
